# Combination of common mtDNA variants results in mitochondrial dysfunction and a connective tissue dysregulation

**DOI:** 10.1073/pnas.2212417119

**Published:** 2022-11-02

**Authors:** Patrick M. Schaefer, Leonardo Scherer Alves, Maria Lvova, Jessica Huang, Komal Rathi, Kevin Janssen, Arrienne Butic, Tal Yardeni, Ryan Morrow, Marie Lott, Deborah Murdock, Angela Song, Kierstin Keller, Benjamin A. Garcia, Clair A. Francomano, Douglas C. Wallace

**Affiliations:** ^a^Center for Mitochondrial and Epigenomic Medicine, Children’s Hospital of Philadelphia, Philadelphia, PA 19104;; ^b^Department of Biomedical Informatics, Children’s Hospital of Philadelphia, Philadelphia, PA 19104;; ^c^The Bert Strassburger Metabolic Center, Sheba Medical Center, Ramat Gan 52561, Israel;; ^d^Department of Biochemistry and Molecular Biophysics, Washington University School of Medicine, St. Louis, MO 63110;; ^e^Department of Medical and Molecular Genetics, Indiana University School of Medicine, Indianapolis, IN 46202;; ^f^Department of Pediatrics, Division of Human Genetics, Perelman School of Medicine, University of Pennsylvania, Philadelphia, PA 19104

**Keywords:** mtDNA haplogroups, mitochondrial disorder, connective tissue disorder, histamine signaling

## Abstract

Mitochondrial dysfunction can be associated with a range of clinical manifestations. Here, we report a family with a complex phenotype including combinations of connective tissue, neurological, and metabolic symptoms that were passed on to all surviving children. Analysis of the maternally inherited mtDNA revealed a novel genotype encompassing the haplogroup J - defining mitochondrial DNA (mtDNA) *ND5* m.13708G>A (A458T) variant arising on the mtDNA haplogroup H7A background, an extremely rare combination. Analysis of transmitochondrial cybrids with the 13708A-H7 mtDNA revealed a lower mitochondrial respiration, increased reactive oxygen species production (mROS), and dysregulation of connective tissue gene expression. The mitochondrial dysfunction was exacerbated by histamine, explaining why all eight surviving children inherited the dysfunctional histidine decarboxylase allele (W327X) from the father. Thus, certain combinations of common mtDNA variants can cause mitochondrial dysfunction, mitochondrial dysfunction can affect extracellular matrix gene expression, and histamine-activated mROS production can augment the severity of mitochondrial dysfunction. Most important, we have identified a previously unreported genetic cause of mitochondrial disorder arising from the incompatibility of common, nonpathogenic mtDNA variants.

## Significance

Here we demonstrate a previously unreported class of mitochondrial disease originating from the incompatibility of common mtDNA variants. Until now, clinical evaluation of the mitochondrial DNA only assessed the frequency of individual variants, and common variants were considered nonpathogenic. Our findings emphasize that common mtDNA variants can cause mitochondrial disease when arising on an uncommon mtDNA background. Thus, idiopathic primary mitochondrial disease patients should be checked for novel combinations of otherwise common variants which may contribute to the etiology of the disorder.

Mitochondria are increasingly recognized as integral factors in many pathologies, including diabetes or neuropsychiatric disorders (1). While most components of the mitochondria are nuclear encoded, the maternally inherited mitochondrial DNA (mtDNA) still retains 13 essential subunits of oxidative phosphorylation (OxPhos) as well as the structural RNAs for the mitochondrial protein synthesis machinery (2–4). During human evolution, functional mtDNA variants arose along radiating maternal lineages that resulted in subtle changes in mitochondrial bioenergetics. These permitted humans to adapt to the new environments they encountered as they migrated out of Africa and around the globe. These adaptive variants function within the context of preexisting mtDNA functional variants, resulting in an integrated bioenergetic state. Those variant combinations that proved regionally beneficial became enriched to form geographically constrained groups of related haplotypes, known as haplogroups (5). We have shown that mixture of different mtDNAs in mice is unstable and results in cognitive impairment and an increase in the stress response (6), suggesting incompatibility between otherwise normal mtDNA variants.

Connective tissue disorders are characterized by alterations in the function of the extracellular matrix (ECM), caused by mutation in collagen genes, collagen redox-modifying enzymes, and/or inflammation (7, 8). These ECM factors have been also been associated with mitochondrial function (9–12).

Diabetes and other metabolic disorders are often associated with a remodeling of the ECM, conferring negative effects on the cardiovascular system (13). The ECM remodeling is thought to be the result of an altered metabolic milieu, activation of matrix metalloproteases, and inflammatory response (14, 15). Given the crucial role of mitochondria in regulating the cellular redox state and innate immune response, mitochondrial dysfunction might contribute to idiopathic connective tissue disorders (16).

Here we report a large, two-generation pedigree in which all of the surviving offspring are variably affected by connective tissue, neurological, and metabolic clinical manifestations. This complex phenotype manifests in all eight surviving children, while another seven were lost in utero. We discovered that this pedigree harbors a novel deleterious mtDNA genotype in which the functional variant of one mtDNA haplogroup has arisen de novo on an incompatible mtDNA background, resulting in mitochondrial dysfunction associated with abnormal calcium regulation. The surviving children also inherited a null mutation in the histidine decarboxylase (HDC) gene, thus diminishing histamine production. Since histamine increases cytosolic calcium, the HDC mutation protected the surviving offspring from the mtDNA defect.

## Results

### Family Harbors a Dysfunctional mtDNA Variant.

We evaluated, assembled, and summarized the clinical data for the two-generation pedigree. The mother and all children present with various combinations of connective tissue manifestations, including Chiari malformation, cranial–cervical instability, hypermobility of joints, skin herniation, wound dehiscence, aortic valve disease, ankylosing spondylitis, fatigability, diabetes, and dysautonomic manifestations (*SI Appendix*). The apparent matrilineal transmission of this complex array of clinical manifestations in all eight surviving offspring suggested an mtDNA disorder.

**Fig. 1.**
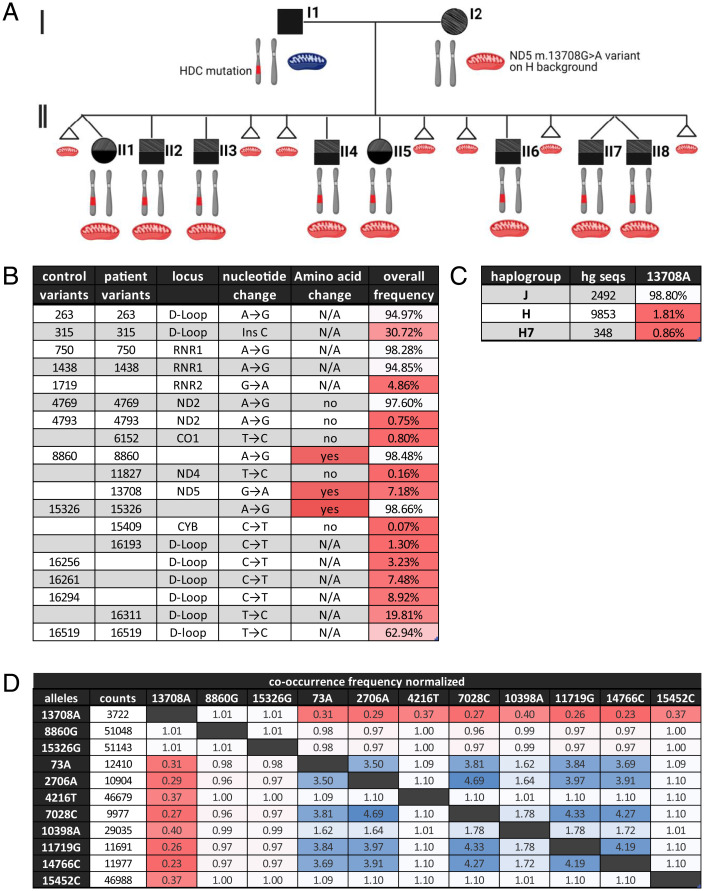
Mitochondrial haplogroup J defining variant *ND5* m.13708G>A on haplogroup H7 background. (*A*) Two-generation pedigree, with squares indicating males, circles indicating females, and triangles indicating miscarriages. Black color indicates Tourette syndrome phenotype; gray shading indicates connective tissue phenotype. Chromosomes indicate the genotype for chromosome 15, with the father and all children being heterozygous for the W317X HDC variant. Red mitochondria indicate maternal mitochondria harboring the *ND5* m.13708G>A variant on haplogroup H7 background. (*B*) Mitochondrial variants found in the proband (mother, I2) and a closely matched control, indicating in which region/gene of the mtDNA the variant is localized, the nucleotide change, the associated amino acid change, and the frequency of the variant in the population (MITOMAP, as of May 4th, 2021). Variants that result in an amino acid change are marked in red; frequencies of the variants are colored white to red, with red indicating rarer variants. (*C*) Frequencies of 13708A in different mtDNA haplogroups colored white to red, with red indicating a lower frequency. Counts display the number of mtDNAs of the respective haplogroup on MITOMAP (as of May 4th, 2021). (*D*) Normalized cooccurrence of mtDNA variants, with values < 1 (red) indicating lower-than-expected cooccurrence within the population and values > 1 (blue) indicating a higher-than-expected cooccurrence (MITOMAP, as of May 4th, 2021). Cooccurrence is displayed for all proband mtDNA variants and variants between mitochondrial haplogroups H and J that result in an amino acid change.

In addition, the children and their father show a range of neurological symptoms (Tourette syndrome, obsessive–compulsive disorder, and autism spectrum disorder [ASD]) that were previously reported to be linked to a paternal, heterozygous mutation in the HDC (W327X) gene (17). Since the father was heterozygous for the normal and the HDC-W327X alleles, we would predict that half of the offspring would have inherited his HDC-W327X chromosome, and half would have inherited his normal HDC gene copy. Since all surviving offspring harbor the HDC-W327X allele and half of the offspring died prenatally, it is likely that those that died inherited the father’s normal HDC allele. Our resulting interpretation of the two-generation pedigree is summarized in Fig. 1*A*.

To determine whether there was an mtDNA contribution to this pedigree, we sequenced the mtDNA of the mother and five of her children, their mtDNA variants being presented in Fig. 1*B* (patient variants). The mtDNA haplogroup is H7, but this family’s mtDNA also contains the haplogroup J-defining variant *ND5* m.13708G>A (codon 458 alanine to threonine). This is noteworthy, since the *ND5* m.13708G>A is very rarely associated with haplogroup H7 (AF = 0.86%, 3/348) in MITOMAP (18) and rarely found on other haplogroup H mtDNAs (Fig. 1*C*). We performed a cooccurrence analysis of all variants of the patient family and variants between mitochondrial haplogroups H and J that result in an amino acid change (Fig. 1*D*). This provides the frequency of the cooccurrence of mtDNA variants in the human population normalized to the expected, randomized frequency calculated according to their abundance. We found that 13708A cooccurs less than expected (from a statistical point of view) with multiple haplogroup H variants (Fig. 1*D*, frequencies of <1), suggesting a possible natural selection against these combinations. The combination of the *ND5* m.13708G>A mutation plus the haplogroup H7 background will here be designated the mtDNA “mutant.”

**Fig. 2.**
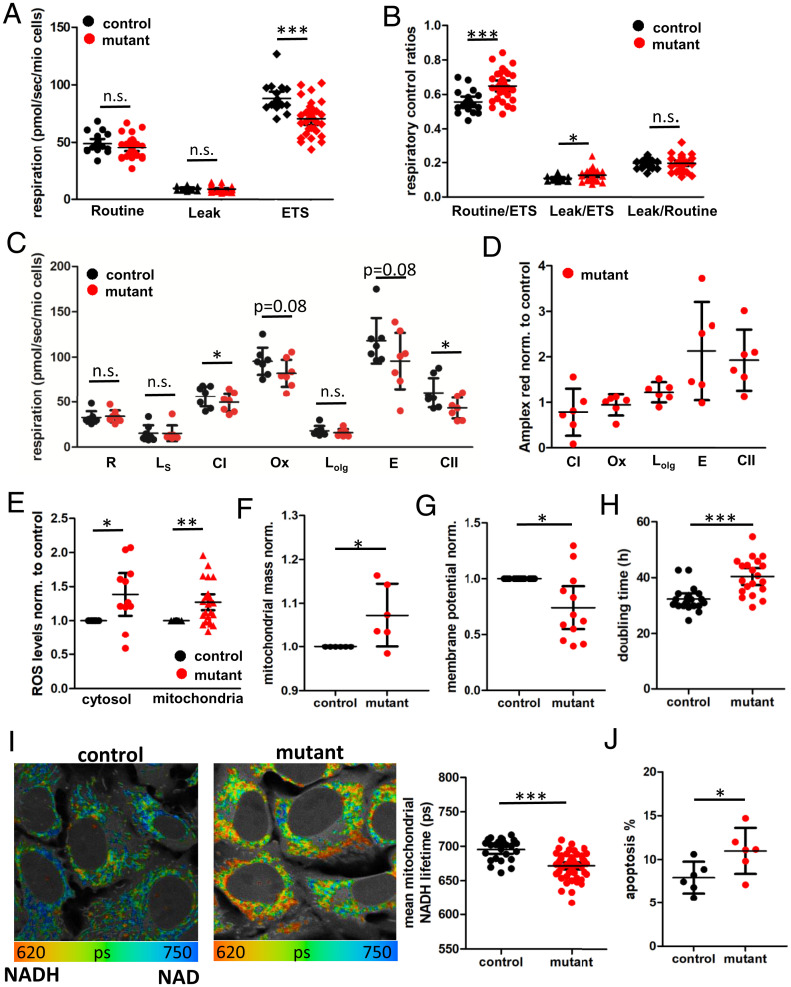
*ND5* m.13708G>A-H7 mtDNA reduces bioenergetic function. (*A* and *B*) Respirometry (*A*) and respiratory control ratios (*B*) of intact transmitochondrial cybrids harboring control (black) or patient (mutant, red) mitochondria (control: *n* = 19, mutant *n* = 31, technical duplicates, Mann–Whitney test). (*C*) High-resolution respirometry in permeabilized cybrids (*n* = 7, technical duplicates, Wilcoxon signed rank test). (*D*) ROS production in permeabilized cybrids quantified as the increase in Amplex red fluorescence at different respiratory states normalized to control (*n* = 6, technical duplicates, one-sample *t* test). (*E*) Cytosolic and mitochondrial ROS levels in control and mutant cybrids quantified as the fluorescence intensity of DCDFA (cytosol, *n* = 11 in technical duplicates, paired *t* test) or Mitosox (mitochondrial, *n* = 26 in technical duplicates, paired *t* test) normalized to control. (*F*) Mitochondrial mass of control and mutant cybrids quantified as fluorescence intensity of MitoTracker CMXRos in flow cytometry normalized to control (*n* = 6, technical triplicates, paired *t* test). (*G*) Mitochondrial membrane potential in control and mutant cybrids measured as red/green fluorescence of JC-1 quantified by flow cytometry normalized to control (*n* = 12, technical duplicates, paired *t* test). (*H*) Doubling time of control and mutant cybrids (*n* = 20, Wilcoxon signed rank test). (*I*) FLIM of mitochondrial NADH in control and mutant cybrids. False-color coding of NADH fluorescent lifetime, with blue indicating a longer lifetime (more oxidized NAD^+^/NADH) and red indicating a shorter lifetime (more reduced NAD^+^/NADH). Gray indicates nonmitochondrial autofluorescence. Quantification of the mean mitochondrial NADH lifetime (*n* = 5, with five image sections and >25 cells/independent experiment, unpaired *t* test). (*J*) Apoptosis in cybrids quantified as the fluorescence intensity of the cells after Annexin V staining using flow cytometry (*n* = 6, technical duplicates, paired *t* test). Error bars display 95% CIs. Significances are indicated by stars, with n.s. = *P* > 0.05, * = *P* < 0.05, ** = *P* < 0.01, *** = *P* < 0.001.

To assess whether the *ND5* m.13708G>A variant on haplogroup H7 affects mitochondrial function, we created transmitochondrial cybrids that have the same 143B(TK^−^) cell nuclear DNA (nDNA) but harbor either the patient mtDNA (mutant) or the mtDNA of a haplogroup-matched normal control (control) (Fig. 1*B*). We observed a lower electron transport system (ETS) capacity with reduced complex I and II respiration in the mutant cybrids (Fig. 2 *A*–*C*). In line with the lower respiration, the mutant cybrids displayed a reduced mitochondrial membrane potential (Fig. 2*G*) and NAD^+^/NADH redox ratio (Fig. 2*I*). In addition, both mitochondrial and cytosolic reactive oxygen species (ROS) levels were increased (Fig. 2 *D* and *E*), as were mitochondrial mass (Fig. 2*F*), doubling time (Fig. 2*H*), and apoptosis (Fig. 2*J*) in the mutant cybrids. In contrast, haplogroup J cybrids harboring the *ND5* m.13708G>A variant in its standard context showed similar respiration compared to haplogroup H cybrids and even reduced ROS production (*SI Appendix*, Fig. S1). Hence, when arising in the wrong context, the *ND5* m.13708G>A variant is incompatible with the resident haplogroup H7 mtDNA functional variants, resulting in mitochondrial dysfunction and clinical manifestations.

**Fig. 3.**
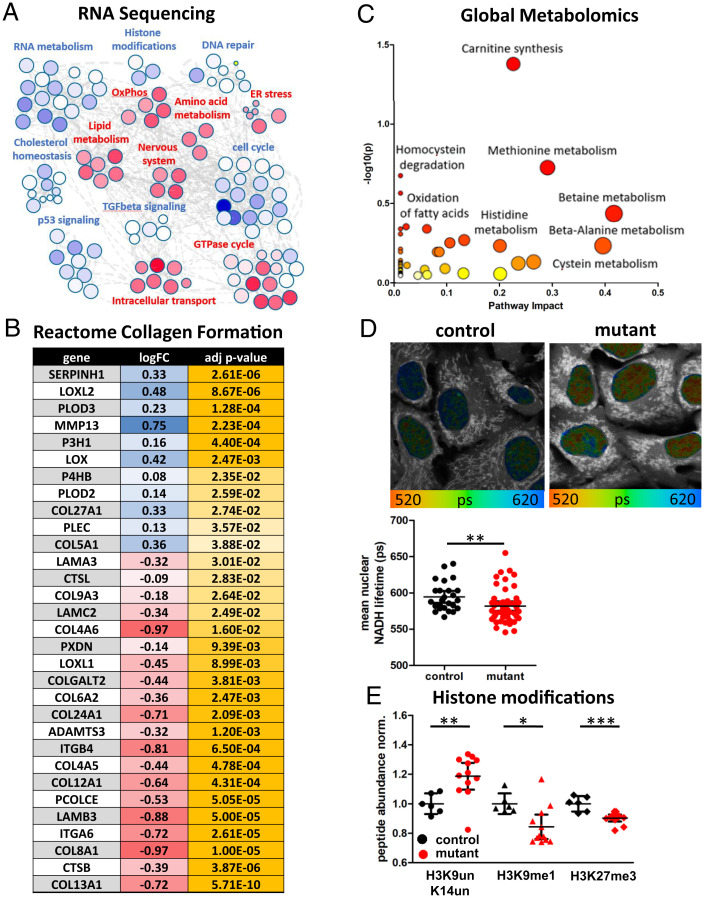
*ND5* m.13708G>A-H7 mtDNA profoundly alters metabolite and expression profile. (*A*) Cluster analysis from GSEA of RNAseq in mutant versus control cybrids using Cytoscape. Every node represents a significant pathway, with blue indicating up-regulation and red indicating down-regulation of the pathway in variant versus control. (*B*) Differential gene expression between mutant and control cybrids for the Reactome Collagen Formation pathway. Log fold change (LogFC) is colored blue–white–red, with blue indicating up-regulation and red indicating down-regulation of a gene in mutant cybrids compared to control. Adjusted (adj.) *P* values are colored white–yellow, with yellow indicating stronger significance. (*C*) Metabolic pathway analysis of global untargeted metabolomics using significantly (*P* < 0.05) altered metabolites between mutant and control cybrids. Size of the dots indicates pathway impact (*x* axis), and color (white–yellow–red) indicates *P* value, with red corresponding to higher significance. (*D*) FLIM of nuclear NADH in control and mutant cybrids. False-color coding of NADH lifetime, with blue indicating a longer NADH fluorescent lifetime and a more oxidized NAD+/NADH redox ratio and red indicating a shorter NADH fluorescent lifetime or more reduced redox ratio. Gray indicates nonnuclear autofluorescence. Quantification of the mean nuclear NADH lifetime revealed a more reduced redox ratio in mutant cybrids (*n* = 5, with five image sections and >25 nuclei/independent experiment, unpaired *t* test). (*E*) Selected histone modifications of histone 3 in control and mutant cybrids displayed as peptide abundance normalized to control (*n* = 6, 12, unpaired *t* test). Significances are indicated by stars, with * = *P* < 0.05, ** = *P* < 0.01, *** = *P* < 0.001.

To further evaluate the impact of the *ND5* m.13708G>A-H7 mtDNA mutant on cellular physiology, we performed multiomics on the cybrids. This revealed profound changes to the gene expression, histone acetylation, and metabolite profile (Fig. 3).

RNA sequencing (RNAseq) revealed down-regulation of OxPhos and nicotinamide metabolism in mutant cybrids (Fig. 3*A* and *SI Appendix*, Fig. S2). This goes along with a more reduced NAD^+^/NADH redox ratio (Fig. 3*D*) and altered nucleotide levels and tricarboxylic acid (TCA) cycle intermediates in the mutant cybrids (*SI Appendix*, Fig. S2 *B* and *C*). Interestingly, while nicotinamide levels were increased, 6-methylnicotinamide was significantly decreased in the mutant cybrids (*SI Appendix*, Fig. S2*B*), indicating a problem in the S-adenosylmethionine (SAM)-dependent degradation of nicotinamide. Global metabolomics point to alterations in methionine metabolism and homocysteine degradation (Fig. 3*C*), and RNAseq revealed a down-regulation of the one-carbon metabolism (Fig. 3*A* and *SI Appendix*, Fig. S2*D*). Targeted metabolomics verified reduced homocysteine levels but increased cystathionine levels and trends toward a reduced SAM/SAH (S-adenosylhomocystein) and GSSG/GSH (oxidized to reduced glutathione) ratio in the mutant cybrids (*SI Appendix*, Fig. S2*E*), revealing a defect in the mitochondrial one-carbon metabolism. Consistent with lower methyl-donor availability, we detected histone hypomethylation in the mutant cybrids (Fig. 3*E* and *SI Appendix*, Fig. S3). In addition, global metabolomics revealed increased levels of acylcarnitines (Fig. 3*C* and *SI Appendix*, Fig. S4*C*) in the mutant cybrids, indicating alterations in fatty acid metabolism. This is further supported by significant changes to the ketone body metabolism and an up-regulation of the cholesterol biosynthesis pathway in RNAseq (*SI Appendix*, Fig. S4 *A* and *B*). Importantly, RNAseq revealed significant differential expression of genes associated with collagen formation and neurodevelopment in the mutant cybrids (Fig. 3 *A* and *B*), linking the mitochondrial variant with the connective tissue phenotype and the neurological symptoms of the family.

Taken together, the *ND5* m.13708G>A variant on the haplogroup H7 background affects collagen metabolism in association with changes in mitochondrial metabolism and redox status, fatty acid metabolism, one-carbon metabolism, and nicotinamide metabolism, thus linking a change in mitochondrial metabolism with ECM structure and function.

### HDC Variant Acts as a Beneficial Nuclear Modifier via Calcium Homeostasis.

Next, we asked whether the heterozygous HDC variant from the father could have a beneficial effect on the maternal *ND5* m.13708G>A-H7 mtDNA, thus explaining why all living offspring carry the HDC variant while seven others died in utero. Given the central role of HDC in histamine signaling (19) and subsequent calcium release from the endoplasmatic reticulum (ER) (20), we assessed calcium homeostasis in the cybrids.

**Fig. 4.**
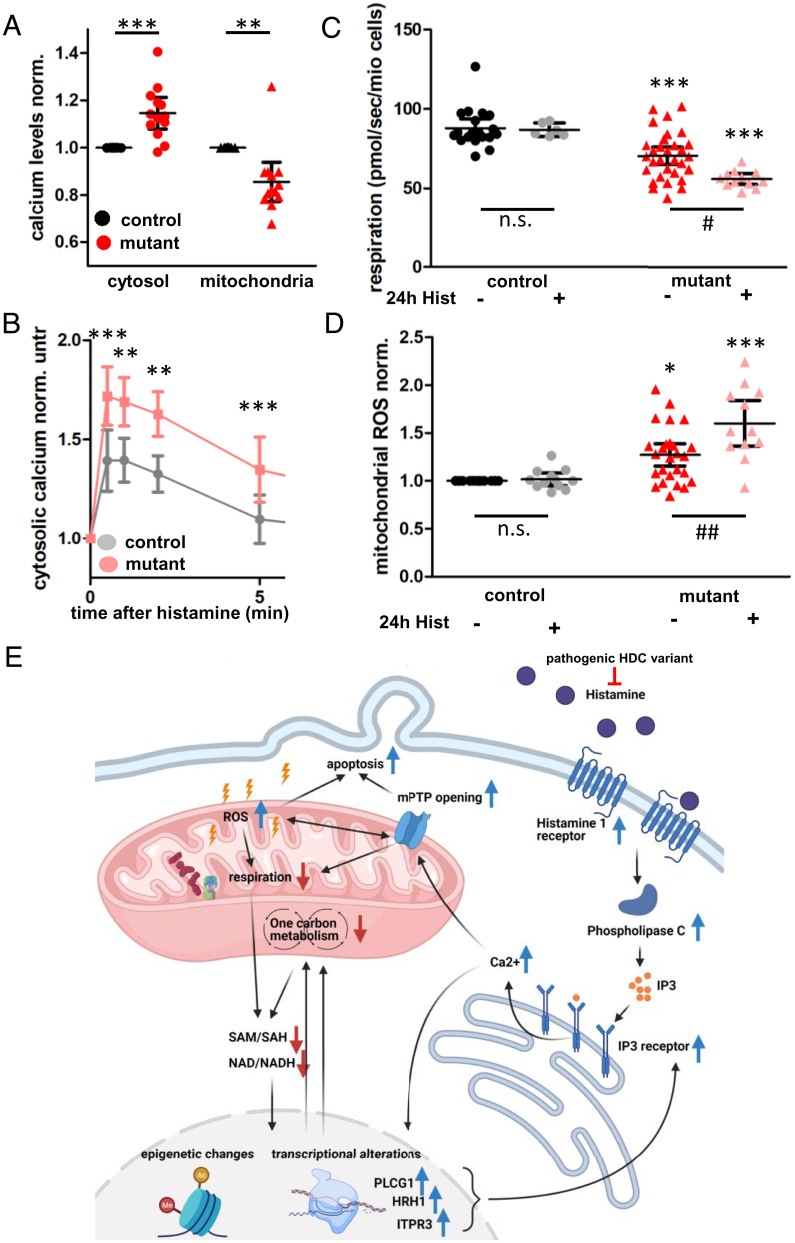
Calcium homeostasis links paternal HDC variant with the *ND5* m.13708G>A-H7 mtDNA. (*A*) Cytosolic and mitochondrial calcium levels in control and mutant cybrids normalized to control measured by confocal microscopy or flow cytometry using Fura Red (cytosol, *n* = 6 microscopy, *n* = 7 flow cytometry, paired *t* test) or Rhod-2 (mitochondria, *n* = 13 flow cytometry, paired *t* test). (*B*) Cytosolic calcium levels in mutant and control cybrids in response to a 100-µM histamine stimulus, normalized to calcium levels preaddition (time point zero). Measured using Fura Red and confocal microscopy (*n* = 7, paired *t* test). (*C* and *D*) ETS capacity (*C*) and mitochondrial ROS levels (*D*) of control and mutant cybrids with and without pretreatment with 100 µM histamine for 24 h. Significances between mutant and control (*) and between treatments (#) were calculated using the Kruskal–Wallis test (*n* = 19, 6, 31, 12, technical duplicates) for respirometry and one-way ANOVA (*n* = 26, 12, 26, 12, technical duplicates) for ROS. (*E*) Diagram visualizing the interaction between a pathogenic variant in the HDC and the *ND5* m.13708G>A-H7 mtDNA. Blue arrows indicate an increase and red arrows a decrease in the mutant cybrids. The *ND5* m.13708G>A-H7 mtDNA reduces respiration and one carbon metabolism, resulting in epigenetic and transcriptional changes that include an up-regulation of Histamine 1 receptor (HRH1), Phospholipase C (PLCG1), and IP3 receptor (ITPR3). Upon histamine, this leads to increased calcium release from the ER, mitochondrial calcium overload, mPTP opening, and apoptosis. The HDC variant reduces the formation of histamine from histidine, thereby preventing histamine toxicity. Created with BioRender.com. Significances are indicated by stars, with n.s. = *P* > 0.05, * = *P* < 0.05, ** = *P* < 0.01, *** = *P* < 0.001.

We found higher cytosolic calcium but reduced mitochondrial calcium levels in the mtDNA mutant cybrids at baseline (Fig. 4*A*), the latter likely due to the reduced mitochondrial membrane potential (Fig. 2*G*). Histamine-induced calcium release from the ER resulted in a stronger increase of cytosolic calcium in the mutant cybrids (Fig. 4*B*), which was weakened but retained upon pretreatment with a mitochondrial calcium uptake inhibitor (*SI Appendix*, Fig. S5*C*) but not after thapsigargin-induced calcium release (*SI Appendix*, Fig. S5*D*). This demonstrates an increased histamine sensitivity in the mutant cybrids, which corresponds to an up-regulation of the histamine 1 receptor signaling pathway on the RNA level (*SI Appendix*, Fig. S5*E*). At the same time, the partial rescue with the mitochondrial calcium uniporter (MCU) inhibitor indicates a lower mitochondrial calcium uptake in the mutant cybrids. This is confirmed by a lower increase of mitochondrial calcium upon histamine treatment (*SI Appendix*, Fig. S5*B*) and down-regulation of the mitochondrial calcium ion transport (*SI Appendix*, Fig. S5*A*) in the mutant cybrids. Taken together, the *ND5* m.13708G>A variant on haplogroup H7 background alters calcium homeostasis and histamine signaling, providing a link to the HDC variant.

To simulate an increased HDC activity, we exposed the cybrids to histamine for 24 h. The m.13708G>A-H7 mutant but not control cybrids showed a further reduced respiration (Fig. 4*C* and *SI Appendix*, Fig. S6*A*) and increased ROS production (Fig. 4*D*) after treatment with 100 µM histamine for 24 h. This could be blocked by simultaneous treatment with the histamine 1 receptor antagonist pyrilamine (*SI Appendix*, Fig. S6 *C–E*). In addition, we found an increased mitochondrial permeability transition pore (mPTP) opening in the mutant cybrids (*SI Appendix*, Fig. S6*B*), indicating that a mitochondrial calcium overload induced via histamine 1 receptor–mediated calcium release from the ER contributes to mutant cybrid cell death (Fig. 4*E*).

If histamine treatment worsens the mitochondrial function in the m.13708G>A-H7 mutant cybrids via the histamine 1 receptor, we checked whether inhibition of the histamine receptor could rescue the mitochondrial function in mutant cybrids. Indeed, pyrilamine inhibition of the histamine 1 receptor mitigated the mitochondrial phenotype between mutant and control cybrids with respect to doubling time (*SI Appendix*, Fig. S7*A*), respiration (*SI Appendix*, Fig. S7*B*), ROS (*SI Appendix*, Fig. S7 *C* and *D*), membrane potential (*SI Appendix*, Fig. S7*E*), calcium homeostasis (*SI Appendix*, Fig. S7 *F* and *G*), and cell death (*SI Appendix*, Fig. S7*H*). In summary, lower histamine levels due to the paternal HDC variant mitigate the increased calcium overload toxicity for the *ND5* m.13708G>A-H7 background, thus explaining why all of the surviving offspring inherited the HDC mutant allele.

The remaining question is why the mother, who does not carry the HDC variant, was affected with the metabolic and connective tissue disorders but was still alive. One possibility is that the mother harbored a nuclear genotype that was protective of the *ND5* m.13708G>A-H7 variant mitochondrial defect. To assess whether the mother’s genotype affected her mitochondrial physiology, we created immortalized lymphoblasts from the mother, four of her children, and three haplogroup-matched controls. For the mother and her children, we found no changes in respiratory capacity or calcium handling compared to controls, although a switch from complex I to complex II respiration and increased cytosolic ROS was observed (*SI Appendix*, Fig. S8 *A–D*). This mild mitochondrial phenotype compared to the mutant cybrids might be explained by low HDC and histamine 1 receptor expression and the presence of the mutant HDC in the patient lymphoblasts as compared to the cybrids (*SI Appendix*, Fig. S7).

Comparing the mother’s mitochondrial physiology to that of her children (*SI Appendix*, Fig. S8 *E–H*), we found that the lymphoblasts of all children but not the mother had increased ROS levels compared to control lymphoblasts (*SI Appendix*, Fig. S8*G*). Since increased ROS predisposes to apoptosis, the mother may harbor a multiple-allele antioxidant genotype not transmitted to her offspring.

To verify whether lowering ROS could mitigate the mother’s phenotype, we treated the *ND5* m.13708G>A-H7 cybrids with *N*-acetylcysteine (NAC) and nicotinamide riboside (NR). Both partially rescued the mitochondrial defect in the mutant cybrids, resulting in a shorter doubling time, higher respiration, and lower cytosolic ROS levels (*SI Appendix*, Fig. S9). This not only supports the mother harboring an enhanced antioxidant genotype but also suggests new therapeutic approaches for this family.

## Discussion

We analyzed an unusual family with mitochondrially inherited mitochondrial dysfunction, neuropsychiatric manifestations, metabolic disruption, and connective tissue manifestations. In identifying an out-of-context mtDNA mutant in the family, we gained three crucial insights into the diversity of mitochondrial disorders.

First, we could establish that a common haplogroup J-defining mtDNA variant causes mitochondrial dysfunction when combined with haplogroup H7, while the same variant in haplogroup J does not affect bioenergetics. While all of the factors which contribute to the adverse effects of this family’s mtDNA are not yet known, one possible factor is the combination of the macrohaplogroup N and haplogroup H7 *ND3* m.10398G>A with the newly acquired haplogroup J *ND5* m.13708G>A variant. In haplogroup J, the *ND5* m.13708G>A variant coincides with the alternative *ND3* allele, *ND3* m.10398A>G, a combination that might result in a complex I defect (Fig. 1*C*). However, this may not be the only factor, since there is the small subhaplogroup J1c8 (59/56,895 sequences on MITOMAP) that harbors the *ND5* m.13708G>A + *ND3* m.10398G>A combination and has not shown a marked mitochondrial defect in a previous study (21, 22). While the exact molecular basis of the intramtDNA *ND5* m.13708G>A haplogroup H7 incompatibility is unknown, one might hypothesize that mitochondrial electron transfer is hampered, resulting in lower respiration, membrane potential, and higher ROS production, all of which predispose to apoptosis. Incompatibility between haplogroup-associated mtDNA variants may explain why the mtDNA is strictly maternally inherited, thus blocking interhaplogroup recombination and the mixing of incompatible variant alleles (2). The existence of functional alterations in mtDNAs has been documented when mtDNAs of different origins have been mixed within a mouse and when different mtDNAs are placed on the same nDNA background (6, 23–25). Likewise, it has been shown that rare combinations of nonpathogenic mtDNA missense mutations in the *ND4* and *ND6* genes are sufficient to cause Leber’s hereditary optic neuropathy (26). On a population level, “out-of-place” mtDNA variants have been reported as risk factors for Parkinson’s disease (27), further supporting our finding. Taken together, we demonstrate that cross-mtDNA mixing between common mtDNA variants can result in incompatibility and mitochondrial disorders. Clinically, this implies the need for not only checking the frequency of mtDNA variants but also the frequency of cooccurrence with otherwise common mtDNA variants when evaluating mtDNAs for pathological relevance. In summary, this provides strong evidence for an incompatibility of common mtDNA variants in humans, resulting in a pathological phenotype.

Second, we demonstrate a mitochondrial–nuclear interaction between the paternal HDC variant and the maternal mtDNA, by establishing an important role for histaminergic signaling in modifying the mitochondrial phenotype. We showed that the HDC null allele (W317X) acts as a beneficial nuclear modifier for the deleterious maternal mtDNA. Given the crucial role for calcium signaling in apoptosis as well as embryogenesis (28), it seems plausible that the seven miscarriages were a result of a noncompensated dysregulation in calcium homeostasis due to the presence of the maternal mtDNA but absence of the HDC variant.

While the mother herself does not harbor the HDC variant, we found lower cytosolic ROS levels in her lymphoblastoid cells compared to those of her children, suggesting the presence of additional nuclear antioxidant modifiers. Since any single modifier would have been passed on to half of the children, it is possible that the mother is homozygous for a rare protective variant and that the single allele the children inherited does not provide sufficient ROS protection. Consistent with previous reports for mitochondrial dysfunction in cells (29) or mice (30), lowering the ROS levels in the *ND5* m.13708G>A-H7 cybrids rescued the mitochondrial phenotype. This explains why all eight living children carry the HDC variant, suggesting that the seven additional miscarriages inherited the father’s normal HDC allele.

Third, we demonstrated that mtDNA variants causing mitochondrial dysfunction can alter the nuclear epigenome and the expression of multiple nuclear coded ECM genes, including multiple collagen isoforms. It has been shown that collagen VI deficiency results in mitochondrial dysfunction, including calcium deregulation and mPTP opening in mice causing a form of muscular dystrophy (31). Interestingly, mPTP desensitization improved the muscular dystrophy in collagen VI mutant cells and mice (32). Mechanistically, it has been suggested that mitochondria could regulate collagen metabolism via the redox state and/or calcium modulation (7), which were significantly altered in the patient cybrids. This hypothesis is supported by the beneficial effect of NR in the patient cybrids, suggesting NAD+ supplementation as a possible therapeutic option. In addition, mitochondria can affect the ECM through ROS production and associated triggering of the stress and inflammatory response (33). Accordingly, reducing ROS levels using NAC improved the mitochondrial defect in the cybrids, and the mother showed lower ROS levels compared to her children, potentially mitigating the absence of the dysfunctional HDC variant. Thus, reducing ROS might be another therapeutic strategy for these patients.

With respect to the underlying cause of the neurological phenotypes in this family, the HDC variant most likely contributes to the neurological phenotype (17), although mtDNA defects are also known to cause neurological disease as well as metabolic phenotypes. For example, a single mtDNA base substitution mutation is sufficient to cause ASD-like symptoms in mice (30), and mitochondrial dysfunction is thought to be a key feature in neurodegenerative disorders (34). That the HDC variant is contributory to the neurological phenotypes is supported by the fact that the father but not the mother displays a Tourette phenotype and by additional studies in recent years that linked the HDC variant to Tourette syndrome (35, 36). However, the skewed inheritance of the HDC gene in the surviving offspring is likely due to the functional interaction with the mtDNA genotype via calcium homeostasis. RNAseq analysis of the patient transmitochondrial cybrids identified the dysregulation of genes associated with neurodevelopment and axon guidance. In addition, we showed that histamine aggravates the mitochondrial phenotype in vitro. Given the prominent role of histaminergic signaling in the brain, it is possible that both the HDC variant and the mtDNA variant contribute to the neuropsychological disorders in the offspring.

In summary, we have demonstrated that incompatibility between common mtDNA variants can result in systemic mitochondrial dysfunction, alteration in the epigenome, and altered expression of an array of ECM genes. Since the maternally inherited mtDNA sequence can only change by the sequential accumulation of mutations arising on mtDNA lineages, each in total linkage disequilibrium, a new functional variant that arises will be tested by natural selection for its compatibility with the functional variants already present in the background mtDNA. If the new variant decreases the OXPHOS efficiency, the new combination will be eliminated by purifying selection, which, in humans, is disease. Because of the high mtDNA mutation rate, the same mtDNA variants have been seen to repeatedly arise on different mtDNA backgrounds, making novel combinations of functional mtDNA variants common. While incompatible combinations will be common in the clinic, natural selection will mean they are rare in the global population.

It is common practice in the clinic and in diagnostic laboratories to test the “pathogenicity” of a patient’s functional mtDNA variant by determining the global allele frequency within our MITOMAP database. However, assessing the frequency of the individual nucleotide variant overlooks the importance of the background on which the variant is found. Therefore, pedigrees with combinations of incompatible but common mtDNA variants may be dismissed as maternally inherited. This can result in erroneous genetic counseling and inherited diseases, such as connective tissue disorders, that previously have not been thought to result from mitochondrial defects but may have a mitochondria etiology.

## Materials and Methods

### Cell Lines and Treatments.

Venous blood samples were obtained from the patient family and three controls following informed consent. This study was approved by the Institutional Review Boards of the University of California, Irvine and Children’s Hospital of Philadelphia. Adult patients consented, in writing, to participate, and parents consented for their children.

Leukocytes were isolated from whole blood by Ficoll-Hypaque gradients, and the isolated cells were Epstein-Barr virus transformed to establish lymphoblast cell lines by Corriell. Lymphoblasts were maintained in Roswell Park Memorial Institute (RPMI) medium 1640 (11879, Gibco) supplemented with 10% heat-inactivated fetal calf serum, 1 mM glucose, and 2 mM uridine. For all lymphoblastoid cell line experimental procedures, 4 million cells were seeded in 15 mL of culture media 48 h prior to the experiment.

Transmitochondrial cybrids were created from the most affected daughter (Fig. 1*A*, II5) and the closest-matching control (female, age range 30 y to 40 y). Lymphoblasts were enucleated with actinomycin D (0.5 µg/mL) for 14 h and then fused with 143B(TK-) ρ^0^ osteosarcoma cells using 45% polyethylene glycol/dimethyl sulfoxide (DMSO). Cells were plated and grown under selection for 4 wk in Dulbecco’s modified Eagle’s medium (DMEM) containing 10% dialyzed fetal bovine serum, 50 µg/mL BrdU, and no uridine (37). Clones were picked and grown separately as individual cell lines, resulting in two clones of patient cybrids harboring the patient mtDNA and one clone of control cybrids harboring the mtDNA of the closest-matching control. Both patient clones were used for the biochemical and bioenergetic comparison to control cybrids, and showed very similar responses. The results were pooled for statistical and graphical representation.

All cybrids were maintained in DMEM (11966, Gibco) supplemented with 10% heat-inactivated fetal calf serum, 1 mM glucose, and 2 mM uridine. For all experimental procedures, 2.5 million cells were seeded in T75 cell culture flasks in 10 mL of culture media 48 h prior to the experiment.

For histamine treatment, a 100-mM histamine stock solution in water was prepared fresh the day of use; 100 µM histamine was added to the cells either 24 h prior or immediately prior to the respective measurements.

For pyrilamine treatment, a 100-mM pyrilamine stock solution in phosphate-buffered saline (PBS) was prepared and stored at −20 °C; 100 µM pyrilamine was added to the cells 24 h prior to the respective measurements and before histamine addition.

For thapsigargin treatment, a 1-mM thapsigargin stock solution in DMSO was prepared and stored at −20 °C; 2 µM thapsigargin was added to the cells immediately prior to the measurements.

For the MCU inhibitor treatment with KB-R7943, a 5-mM KB-R7943 stock solution in DMSO was prepared and stored at −20 °C; 10 µM KB-R7943 was added to the cells 5 min prior to the measurements.

For NR and NAC treatments, the culture media was supplemented with 300 µM NR or 1 mM NAC, and cells were cultured in the continuous presence of both compounds. Representative measurements were started 1 wk after initiation of the treatments and continued for 3 wk. All parameters assessed were checked for trends coinciding with treatment duration, but cellular physiology remained stable.

### mtDNA Sequencing.

The entire mtDNA was PCR amplified in two fragments using the SequalPrep Long PCR Kit (Invitrogen #A10498) with the following primers: 1) hmtL9611: 5′-TCCCACTCCTAAACACATCC-3′, hmtH1405: 5′-ATCCACCTTCGACCCTTAAG-3′; and 2) hmtL1305: 5′-GTAAGCGCAAGTACCCACG-3′, hmtH9819: 5′-GCCAATAATGACGTGAAGTCC-3′. The PCR products were visualized on an agarose gel with ethidium bromide to assess amplification and product size specificity. For library preparation, the two PCR fragments were combined in equal ratios to make 200 ng of DNA, fragmented, and blunt-end ligated to 3′ P1 and barcoded 5′ A-BC adaptors (Life Technologies) using the NEBNext Fast DNA Fragmentation & Library Prep Set for Ion Torrent (New England Biolabs #E6285S/L). Size selection was done using Agencourt AMPure XP beads (Beckman Coulter). Ready amplified libraries were quantified with the Ion Library Quantitation kit (Life Technologies #4468802). Multiple barcoded samples per run were pooled in equimolar ratios, and templated ion sphere particles (ISPs) were prepared and enriched on the One Touch 2 and ES systems using the Ion PGM Template OT2 200 kit (Life Technologies #4480974). The enriched templated ISPs were then loaded on an Ion 318 sequencing chip and sequenced on an Ion Personal Genome Machine (PGM) using Ion PGM Sequencing 200 v2 kit (Life Technologies). Data were analyzed using the Ion Torrent Suite v4.0.1 and NextGENe software v2.3.3. Haplogroups were identified using the plugin MtDNA Variant Caller 4.4.3.3 and the HaploGrep algorithm.

### Doubling Time.

The doubling time was determined in conjunction with the respirometry and flow experiments. Cells were harvested and counted for respirometry/flow cytometry. This allowed calculation of the doubling time via the formula

$$\text{Doubling}\;\text{time}\;\left(\text{h}\right)=\text{culture}\;\text{time}\;\left(\text{h}\right)/{\text{log}}_{2}\left({\text{cell}\;\text{number}}_{\text{end}}{\text{/cell}\;\text{number}}_{\text{start}}\right).$$

### High-Resolution Respirometry in Intact Cells.

Respiration in intact cells was measured as described (38). Briefly, the Oroboros Oxygraph-2k was first calibrated to air with the respective cell culture medium of the cells to be measured (block temperature = 37 °C, stirrer speed = 750 rpm, oxygen sensor gain = 4, and data recording interval = 2 s). Subsequently, the cells were harvested, and 2 million cells, resuspended in their conditioned medium, were loaded into each chamber of the respirometer. The first oxygen consumption plateau corresponds to Routine respiration, determining respiration under endogenous substrates and energy demand. Addition of 0.5 µL of 5 mM oligomycin blocks complex V, resulting in Leak respiration, which determines proton leak across the inner mitochondrial membrane independently of complex V. Stepwise addition of 1 mM carbonyl cyanide p-trifluoro-methoxyphenyl hydrazone (FCCP) reveals the ETS capacity, which determines the maximum respiratory capacity under infinite energy demand. Lastly, 5 µM antimycin A was added to determine background respiration, which is subtracted from the other respiratory states.

Respirometry and parallel ROS production using Amplex UltraRed were performed as described previously (39). Briefly, air calibration was performed using the same settings as for intact cells, but in Mir05 respiratory buffer (0.5 mM egtazic acid (EGTA), 3 mM MgCl_2_, 60 mM lactobionic acid, 20 mM taurine, 10 mM KH_2_PO_4_, 20 mM Hepes, 110 mM D-sucrose, and fatty acid-free bovine serum albumin [1 g/L], and 5 mM Diethylenetriamine pentaacetate). After air calibration, the chambers were closed, and 10 µM Amplex UltraRed (10 mM stock in DMSO), 0.5 U/mL horseradish peroxidase (500 U/mL stock in Mir05), and 5 U/mL superoxide dismutase (5,000 U/mL stock in Mir05) were added. Subsequently, ROS calibration was performed with two consecutive injections of 0.2 µM hydrogen peroxide (Fluorescence-Sensor Green; gain for sensor = 1,000, and polarization voltage = 300 mV).

Cells were harvested, and 2 million cells in 400 µL of Mir05 were added to each chamber. The first plateau displays Routine respiration (R). Cells were then permeabilized by addition of 5 µg/mL digitonin. After addition of 5 mM pyruvate, 2 mM malate, and 10 mM glutamate, cells show a leak respiration (L_S_), and 5 mM adenosine diphosphate resulted in complex I-linked respiration (CI). Further addition of 10 mM succinate revealed OxPhos capacity (Ox), the maximal capacity of mitochondria to produce adenosine 5′-triphosphate upon substrate saturation. Addition of 1.25 µM oligomycin resulted in Leak respiration (L_Olg_), and stepwise addition of FCCP resulted in ETS capacity (E). Addition of 2.5 µM rotenone blocked complex I and revealed complex II-linked respiration (CII). Lastly, 5 µM antimycin A was added to determine background respiration, which is subtracted from the other respiratory states. ROS production at the different respiratory states were first corrected for background flux before addition of cells, and calculated as ROS production/respiration normalized to control.

### Flow Cytometry.

For all flow cytometry experiments, cells were seeded 48 h prior in T75 cell culture flasks in their respective medium. The day of the experiment, cells were harvested and counted, and, for each condition, 1 million alive cells were stained in Tyrodes buffer (135 mM NaCl, 5 mM KCl, 1.8 mM CaCl_2_, 20 mM Hepes, 5 mM glucose, 1 mM MgCl_2_, pH 7.4) with the respective dye. Dependent on the dye, cells were washed in Tyrodes buffer and divided into two technical replicates, and 10,000 cells were measured for each replicate in Tyrodes buffer on an LSR Fortessa (BD). Alive singlets were gated in the forward scatter/side scatter plot, and the mean fluorescence of the respective channel was quantified, corrected for the mean fluorescence of an unstained cell sample in that channel. Mitochondrial localization of the mitochondrial dyes following the respective staining procedures was checked prior to flow cytometry experiments, using confocal microscopy.

### Mitochondrial Mass Using MitoTracker.

For determination of mitochondrial mass, cells were stained with 100 nM MitoTracker Deep Red FM or 100 nM MitoTracker Red CMXRos for 30 min at 37 °C. The mean fluorescence was quantified in the Allophycocyanine (APC) channel (excitation [ex]: 640 nm; emission [em]: 670/14 nm) for MitoTracker Deep Red FM or the mCherry channel (ex: 561nm; em: 610/20 nm) for MitoTracker Red CMXRos.

### Mitochondrial Membrane Potential Using JC1.

For determination of mitochondrial membrane potential, cells were stained with 1 µg/mL JC-1 for 30 min at 37 °C. The mean fluorescence was quantified in the fluorescein isothiocyanate (FITC) channel (ex: 488 nm; em: 530/30 nm) and the phycoerythrin (PE) channel (ex: 561 nm; em: 582/15 nm), and the ratio of PE/FITC was calculated as a surrogate for membrane potential. This was verified by measuring cells treated with 5 µM FCCP upon staining, which showed a lower PE/FITC fluorescence ratio.

### Mitochondrial ROS.

For determination of mitochondrial ROS, cells were stained with 5 µM MitoSOX Red for 15 min at 37 °C. The cells were spun down and resuspended in fresh Tyrodes buffer. The mean fluorescence was quantified in the PerCP channel (ex: 488 nm; em: 695/40 nm).

### Cytosolic ROS.

For determination of cytosolic ROS, cells were stained with 1 µM H2DCFDA for 30 min at 37 °C. The mean fluorescence was quantified in the FITC channel (ex: 488 nm; em: 530/30 nm).

### Mitochondrial Calcium.

For determination of mitochondrial calcium, cells were stained with 10 µM Rhod-2 AM (1 mM stock in DMSO with potassium borohydride) + 0.02% pluronic acid for 30 min at room temperature. The cells were washed once and resuspended in fresh Tyrodes buffer. The mean fluorescence was quantified in the PE channel (ex: 561 nm; em: 582/15 nm).

### Cytosolic Calcium.

For determination of cytosolic calcium, cells were stained with 1 µM Fura Red AM + 0.02% pluronic acid for 30 min at 37 °C. The cells were spun down and resuspended in fresh Tyrodes buffer. The mean fluorescence was quantified in the BV650 channel (ex: 405 nm; em: 655/8 nm) and the PerCP channel (ex: 488 nm; em: 695/40 nm). The ratio of BV650/PerCP was calculated as a surrogate for membrane potential. In confocal microscopy, cells were excited at 405 and 488 nm, and emission was detected between 500 and 758 nm.

### Apoptosis.

For determination of apoptosis, cells were harvested, washed in PBS, and 0.5 million cells were stained in 200 µL of annexin-binding buffer (10 mM Hepes, 140 mM NaCl_2_, 2.5 mM CaCl_2_, pH 7.4) supplemented with either 10 µL of Annexin V Pacific Blue or 10 µL of Annexin V FITC for 15 min at room temperature. After staining, 800 µL of annexin-binding buffer was added, and the cells were measured using flow cytometry. The mean fluorescence was quantified in the Pacific Blue channel (ex: 405 nm; em: 450/50 nm) or the FITC channel (ex: 488 nm; em: 530/30 nm), respectively. No live-cell gate was applied, and the percentage of positive cells was quantified and normalized to control.

### mPTP Opening.

The mPTP opening was measured using the calcein-cobalt method as described (40). Briefly, cells were stained in Kreb’s Ringer buffer (KRB) (135 mM NaCl, 5 mM KCl, 0.4 mM KH_2_PO_4_, 1 mM MgSO_4_, 20 mM Hepes, 5.5 mM glucose, pH 7.4) supplemented with 1 µM calcein, 200 nM MitoTracker Red CMXRos, 2 mM CoCl_2_, and 200 µM sulfinpyrazone for 15 min at 37 °C. Cells were washed once in modified KRB (KBR + 1 mM CaCl_2_), split, and resuspended in modified KRB with and without 1 µM ionomycin (positive control), and measured using flow cytometry. The mean fluorescence was quantified in the FITC channel (ex: 488 nm; em: 530/30 nm) for calcein and the mCherry channel (ex: 561 nm; em: 610/20 nm) for MitoTracker Red CMXRos. The mPTP opening was calculated as the mean calcein fluorescence of cells treated with ionomycin divided by the mean calcein fluorescence of untreated cells and then normalized to control cells.

### Confocal Microscopy.

For all confocal microscopy experiments, 50,000 cybrids were seeded in each quadrant of four-chambered 35-mm dishes with glass bottoms (Cellview, 627870) in their standard culture medium 48 h prior to the measurement. On the day of the experiment, medium was exchanged for the respective staining solution, and cells were imaged in Tyrodes buffer at 37 °C and atmospheric CO_2_ after a 15-min equilibration in the microscope incubation chamber. For lymphoblasts, the four-chambered 35-mm dishes with glass bottoms were precoated with 2.5 µg/cm^2^ Cell-Tak (Corning) for 30 min at 37 °C and rinsed with PBS twice, and 500,000 cells were seeded into each quadrant in Tyrodes buffer immediately prior to the 15-min incubation in the microscope incubation chamber. Imaging was performed using a Zeiss LSM 710 in combination with the Zen 2012 software, for image acquisition and analysis.

### Fluorescence Lifetime Imaging Microscopy of NADH Autofluorescence.

Fluorescence lifetime imaging microscopy (FLIM) of NADH was performed on a laser scanning microscope (Zeiss LSM 710) as described (41). Briefly, NADH was excited using two-photon excitation with a pulsed (80 MHz, 100-fs pulse width) titanium–sapphire laser at a power of <5 mW on the sample. Time-correlated single-photon counting with the hybrid detector HPM-100-40 was performed. The detector was coupled to the Non-Descanned Detection port of the LSM 710. FLIM images (512 × 512 pixel) were taken at a temporal resolution of 256 time channels within a pulse period of 12.5 ns. Final settings were as follows: 60-s collection time; ∼15-µs pixel dwell time; 135 × 135 µm^2^ scanning area, Plan-Apochromat 63×/1.40 Oil DIC M27 lens, 730-nm excitation wavelength, and 460/50 nm band-pass emission filter. Data were recorded using SPCM 9.8 and subsequently analyzed using SPCImage 8.0 assuming a biexponential decay. Final analysis settings were as follows: weighted least squares fit method, lifetime components fixed to 400 ps and 2,500 for free and protein-bound NADH, square binning of two, peak threshold adapted to background, and shift fixed at a pixel of clear NADH signal. The mean lifetime (τ_mean_) was calculated, and fitting of the calculated lifetime curve was confirmed by checking the mean χ^2^, which should be below 1.2. For subcellular analysis, nuclei were selected using region of interests, with at least five nuclei analyzed per image. The mitochondria-rich regions were selected using the threshold function.

### RNAseq and Analysis.

For RNAseq, cybrids were seeded and cultured as for biochemical measurements, and harvested, and a cell pellet of 1.5 million cells was flash frozen and sent to Genewiz (Azenta Life Science) for RNAseq using Poly-A-primers. Eighteen Human Tourette RNA-sequencing Fastq files, consisting of 6 controls and 12 mutant samples, were processed using the STAR alignment (42) tool and subsequently normalized using the RSEM (43) package based upon the hg38 reference genome (44) and the Gencode version 23 gene annotation (45).

Differential gene expression analysis was performed by comparing each gene in the control group vs. the treatment group. The voom procedure (46) was used to normalize the RSEM generated expected counts followed by differential expression testing using R package limma (47) to obtain *P* values and LogFC. Specifically, a total of 58,581 genes were tested for differential expression between the control and treatment samples. Pathway enrichment was performed on control vs. treatment samples using gene set enrichment analysis (GSEA) (48, 49) version 4.1.0 using a weighted scoring scheme and Hallmark and C2 CP gene sets.

All input data and code can be found on GitHub: https://github.com/komalsrathi/tourette-analysis.

### Metabolomics.

For metabolomics, 1.5 million cybrids were seeded in 10-cm cell culture dishes and cultured using standard culture conditions for 48 h. The day of the experiment, cells were transferred on ice into a cold room, medium was removed, cells were rinsed once using ice-cold PBS, and liquid nitrogen was poured directly on the cells. The cell slush was scraped off and transferred into a 1.5-mL tube that was flash frozen in liquid nitrogen and stored at −80 °C until metabolite preparation. A separate aliquot was taken for determination of protein concentration using bicinchoninic acid (BCA).

For untargeted liquid chromatography–mass spectrometry (LC/MS) metabolomics, 50 µL of each thawed cell pellet on ice was aliquoted for untargeted metabolomics, and a 5-µL aliquot of each sample was pooled together and divided to make six 50-µL quality control samples. Methanol (200 µL) was added to each sample, vortexed vigorously for 10 s, and centrifuged at 14,000 rpm for 10 min at 4 °C. Then, 2 × 100-µL aliquots (one for reversed phase C18 LC/MS and one for Hydrophilic Interactin Chromatography (HILIC)/MS) of the supernatant were placed into one of two 96-well plates and dried under nitrogen at 30 °C. Dried samples were reconstituted in 200 µL of High-performance liquid chromatography (HPLC) mobile phases before LC/MS analysis on a Thermo Vanquish UHPLC/Orbitrap ID-X mass spectrometer. Compound Discoverer (Thermo Fisher Scientific) was used to generate principle component analysis plots from the metabolite signals extracted from the raw data files, fold changes, *P* values, heat maps, and whisker plots, and perform a database search for metabolite identification. Pathway analysis was performed in MetaboAnalyst 5.0 (50), using all metabolites that are significantly (*P* < 0.05) up or down in mutant versus control cybrids.

For targeted LC/MS metabolomics of organic acids and malonyl and acetyl CoA, aliquots (100 µL) of frozen cell lysates were homogenized in equal volumes of acetonitrile/0.6% formic acid. Metabolites were extracted in cold aqueous/organic solvent mixtures according to validated, optimized protocols in our previously published studies (51, 52). These protocols use cold conditions and solvents to arrest cellular metabolism and maximize the stability and recovery of metabolites. Each class of metabolites was separated with a unique HPLC method to optimize their chromatographic resolution and sensitivity. Quantitation of metabolites in each assay module was achieved using multiple reaction monitoring of calibration solutions and study samples on an Agilent 1290 Infinity UHPLC/6495 triple quadrupole mass spectrometer (52). Raw data were processed using Mass Hunter quantitative analysis software (Agilent). Calibration curves (R^2^ = 0.99 or greater) were either fitted with a linear or a quadratic curve with a 1/X or 1/X2 weighting.

### Posttranslational Modifications of Histones.

Histone isolation from cybrids was performed as described previously (53). Briefly, cells were lysed in nuclear isolation buffer (NIB; 15 mM Tris, 60 mM KCl, 15 mM NaCl, 5 mM MgCl_2_, 1 mM CaCl_2_, 250 mM sucrose, 1 mM dithiothreitol, 500 µM AEBSF, 5 nM microcystin, 10 mM sodium butyrate) supplemented with 0.3% Nonidet P-40 for 5 min, and nuclei were pelleted and washed three times in NIB. Histones were extracted in 0.2 M sulfuric acid for 4 h and precipitated from the sulfuric acid using 20% TCA. Histone precipitate was rinsed using ice-cold acetone + 0.1% hydrochloric acid, air dried, and resuspended in water, and the concentration was determined using BCA.

For histone mass spectrometry, primary amines and monomethyllysine residues of histones were derivatized using propionic anhydride in acetonitrile and ammonium hydroxide. Histones were dried, then digested using trypsin, and propionylation was performed again to derivatize the newly generated N termini. Samples were dried and then desalted using in-house stage tips. Peptides were separated on an EASY-nLC 1000 using 0.1% formic acid in water as buffer A, 0.1% formic acid in acetonitrile as buffer B, and C18 as trap and analytical stationary phases (54). Data were acquired on a Thermo Q Exactive using DIA and processed in EpiProfile 2.1 (55).

### Quantification and Statistical Analysis.

In all graphs, each data point represents an independent experiment, with the mean and 95% CIs being indicated by the error bars. This information as well as the number of independent experiments and the respective statistical tests are indicated in the figure legends. Statistical analysis was performed using Graph Pad Prism 5. Independent experiments for respirometry and flow cytometry are defined as independent experimental procedures on another day and a new batch of cells. Each measurement was performed in technical duplicates (as indicated in the figure legends), and their average (mean) was used as the independent data point. For flow cytometry, the mean fluorescence intensity/intensity ratios were normalized to control for every independent experiment. For microscopy, independent measurements (data points) were defined as images from independent wells with, maximally, five images per independent experiment (day and cell batch). For RNAseq, metabolomics, and histone modifications, independent experiments indicate samples collected on different days and from different cell batches. Statistical analysis for RNAseq, metabolomics, and histone modifications are described in *Materials and Methods*. Significances are indicated by (*) or (#) and represent *P* values of *= *P* < 0.05, ** = *P* < 0.01, *** = *P* < 0.001 throughout the manuscript.

## Supplementary Material

Supplementary File

## Data Availability

Anonymized RNAseq data discussed in this publication have been deposited in NCBI's Gene Expression Omnibus (56) and are accessible through GEO Series accession number GSE215859 (https://www.ncbi.nlm.nih.gov/geo/query/acc.cgi?acc=GSE215859) (57). Metabolomics data have been deposited in MetaboLights (58) and are accessible through MetaboLights accession number MTBLS4253 (https://www.ebi.ac.uk/metabolights/MTBLS4253/descriptors) (59). All other study data are included in the article and/or *SI Appendix*.
