# Supplementary material for: Combination of common mtDNA variants results in mitochondrial dysfunction and a connective tissue dysregulation
Source: Proc Natl Acad Sci U S A. 2022 Nov 2;119(45):e2212417119. doi: 10.1073/pnas.2212417119 (PMC9659340; doi:10.1073/pnas.2212417119)
Supplement: Supplementary File [file pnas.2212417119.sapp.pdf]

PNAS
www.pnas.org

Supplementary Information for

Combination of Common mtDNA Variants Results in Mitochondrial Dysfunction and a Connective Tissue Dysregulation

Patrick M. Schaefer,[a] Leonardo Scherer Alves,[a] Maria Lvova,[a] Jessica Huang,[a] Komal Rathi,[b] Kevin Janssen,[a] Arrienne Butic,[a] Tal Yardeni,[a,c] Ryan Morrow,[a] Marie Lott,[a] Deborah Murdock,[a] Angela Song,[a] Kierstin Keller,[a] Benjamin A. Garcia,[d] Clair A. Francomano,[e] Douglas C. Wallace[a,f*].

[a] Center for Mitochondrial and Epigenomic Medicine, Children's Hospital of Philadelphia, Philadelphia, PA, 19104;
[b] Department of Biomedical Informatics, Children's Hospital of Philadelphia, Philadelphia, PA, 19104;
[c] The Bert Strassburger Metabolic Center, Sheba Medical Center, Ramat Gan, 52561, Israel;
[d] Department of Biochemistry and Molecular Biophysics, Washington University School of Medicine, St. Louis, MO, 63110;
[e] Department of Medical and Molecular Genetics, Indiana University School of Medicine, Indianapolis, IN, 46202; and
[f] Department of Pediatrics, Division of Human Genetics, Perelman School of Medicine, University of Pennsylvania, Philadelphia, PA, 19104

* Douglas C. Wallace, 3501 Civic Center Boulevard, Room 6060, Philadelphia, PA, 19104, USA   Phone: (267) 425-3034
Email: wallaced1@chop.edu

This PDF file includes:

Supplementary text
Figures S1 to S9
SI References

Supplementary Information Text

Patient family and detailed clinical findings

Summation of previous report [1] with augmented familial observations.

I1: Male, age 48: (Tourette syndrome, Obsessive-Compulsive Disorder [OCD]). "history of motor tics including shoulder shrugs and neck stretching. Vocal tics included persistent clearing of the throat" [1].

I2: Female, age 46 (connective tissue, metabolic). "She has no history of tics, OCD, trichotillomania, or Attention Deficit Disorder (ADHD)" [1]. Additional familial reported findings: pseudotumor cerebri (increased pressure around brain), joint hypermobility with post-compression detethering and decompression (tethered cord syndrome: tissue attachments that limit the movement of the spinal cord within the spinal column), chiari malformation (extension of brain into spinal cord, cranio-cervical mobility requiring fusion), hypermobility below cranio-cervical fusion, chronic diarrhea, ketosis, difficulties with temperature regulation, weight loss and nausea on Diamox (carbonic anhydrase inhibitor).

II1: Female, age 25 (Tourette syndrome, OCD, metabolic disease). Aspergers (autism spectrum disorder), ADHD, OCD, hair pulling (trichotillomania), skin-picking (dermatillomania) [1]. Additional familial reported findings: Low energy and frequent mild fevers.

II2: Male, age 23 (Tourette syndrome, connective tissue, and metabolic disorders). “Motor tics...” “Vocal tics consisted of throat clearing”. “Type I diabetes, severe myopia, symptomatic chiari malformation, and tethered spinal cord”, “joint hypermobility,..high, narrow palate..” “tricuspid and mitral valve” dysfunction, “diagnosed with a variant of EDS [Ehler-Danlos syndrome]” [1]. Additional familial reported findings: skin herniation which spontaneously improved, possible ankylosing spondylitis (altered posture), kidney issues, DMI (diabetes mellitus insipidus).

II3: Male, age 21 (Tourette syndrome, connective tissue, and cardiovascular disorders). “mild clubbed feet”, mild joint hypermobility”, ‘chiari malformation”, “myopia, bicuspid aortic valve and root dilation”, “sleep apnea” [1]. Additional familial reported findings: Severe cranio-cervical instability, brainstem surgery, aortic aneurism (44mm) with oval aorta on MRI, syncope (loss consciousness) with exercise, supraventricular tachycardia and bradycardia, neurogenic bladder, and patches on retina.

II4: Male, age 18 (Tourette syndrome, connective tissue, and metabolic disorders). “cardiac involvement, pectus excavatum and high arced palate” [1]. Additional familial reported findings: chiari malformation, dysautonomia, and tall (192cm).

II5: Female, age 16 (Tourette syndrome, connective tissue, metabolic and immunological disorders). “Type I diabetes, autonomic dysfunction (tachycardia and narrowed vision), severe joint pain, and symptomatic chiari malformation”, “diagnosed with early-onset osteo-arthritis and a variant of EDS” [1]. Additional familial reported findings: post-compression detethering and decompression, muscle weakness, kidney reflux, cranio-cervical instability, ankylosing spondylitis requiring Lidocaine infusions into spinal cord (assessment “life stinks”), dysautonomia, dehiscence (wound reopening post-surgery), unusual blood vessels brain to pelvis with stroke at age five, altered prothrombin time, multiple drug allergies, multiple infections including methicillin-resistant Staphylococcus aureus, Herpes, and Candida.

II6: Male, age 13 (Tourette syndrome, connective tissue, and metabolic disorders). “evaluated for probable connective tissue disorder with pectus excavatum and hypermobile joints.”: Brain MRI revealed chiari malformation and hydromyelia syrinx”, “surgical … spinal cord detethering”, “ketone utilization disorder” [1]. Additional familial reported findings: episodic bradycardia, 3-β-keto-thiolase deficiency, high prolactin levels, carnitine bicitrate therapy.

II7: Male, age 10, monozygotic twin (Tourette syndrome and OCD, connective tissue, and metabolic disorders). “symptomatic chiari malformation”, “high/arched palate, bilateral dislocation of hips, and moderate joint laxity”, “mild convex cervical scoliosis centered at C6/C7”, “sleep apnea and breathing pauses while awake”, “possible connective tissue disorder at 6 years of age”, “an EDS variant” [1]. Additional familial reported findings: pseudotumor cerebri (increased pressure around brain) treated with ventriculoperitoneal shunt, left eye ptosis, joint hypermobility, post-compression detethering, and decompression, frequently falls asleep, chronically elevated CO2 while sleeping, apnea-hypoapea greater than 10 second, altered prothrombin time.

II8: Male, age 10, monozygotic twin (Tourette syndrome, connective tissue, cardiovascular and metabolic disorders).” described as having thin skin, easy bruisability, and joint laxity and dislocation. He has been diagnoses with a tethered spinal cord but has not required surgery”, “bilateral strabismus with surgical correction” [1]. Additional familial reported findings: prediabetic (blood sugar 110-112 mg/dL); hypermobile joints with subluxation (partial bone dislocation on CT) of knees, hips, and ankles with severe foot and leg pain; superficial veins on left arm; altered prothrombin time; left ventricular hypertrophy and hypertension starting at 8 years.

Supplementary Figures

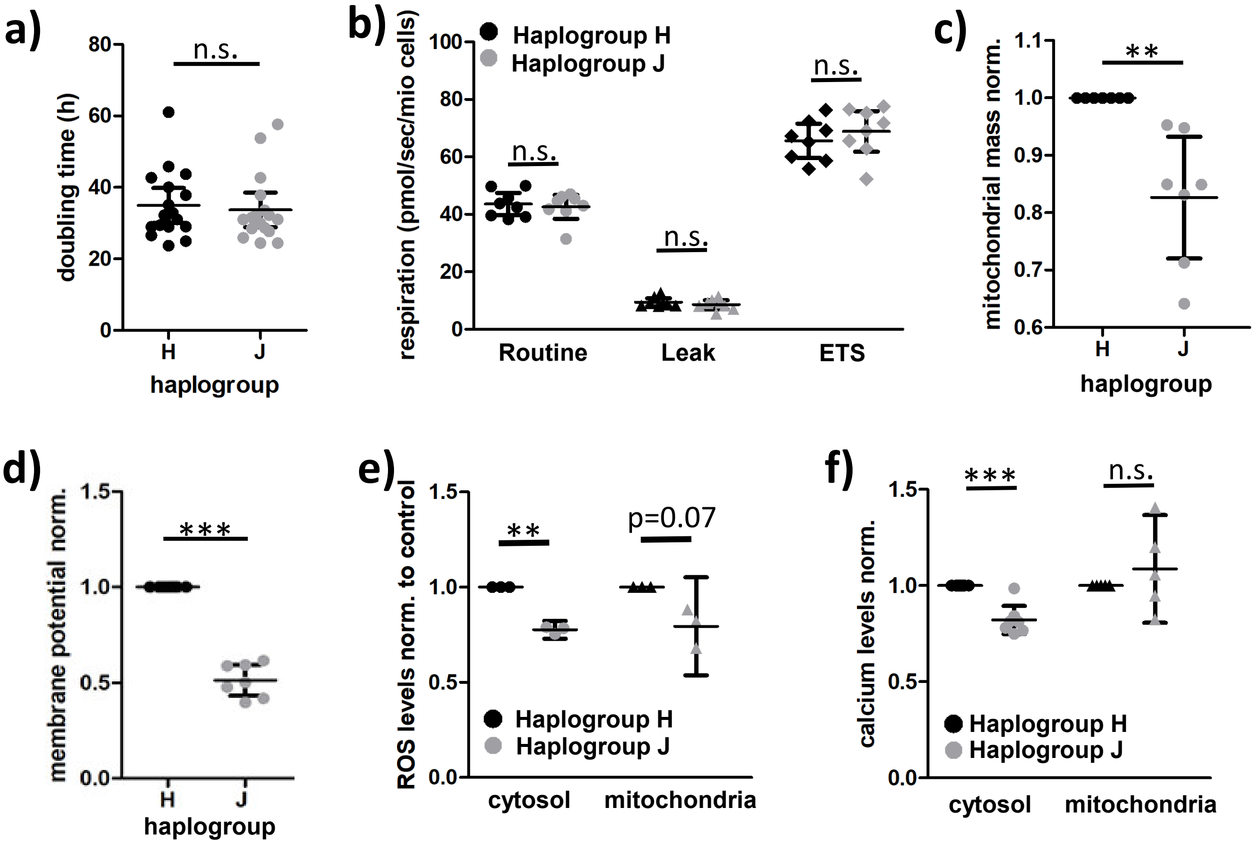

Fig.S1 Mitochondrial haplogroup J shows similar bioenergetics to haplogroup H
a) Doubling time of haplogroup H and J cybrids in low glucose media (n = 17, Mann Whitney test). **b)** High resolution respirometry of intact haplogroup H (black) or J (grey) cybrids (n = 8, each in technical duplicates, Mann Whitney test). **c)** Mitochondrial mass of haplogroup H and J cybrids quantified as fluorescence intensity of Mitotracker Deep Red in flow cytometry normalized to haplogroup H (n = 6, technical duplicates, paired t-test). **d)** Mitochondrial membrane potential of haplogroup H and J cybrids measured as the ratio of red to green fluorescence of JC-1 quantified by flow cytometry normalized to haplogroup H (n = 7, technical duplicates, paired t-test). **e)** Cytosolic and Mitochondrial ROS levels of haplogroup H and J cybrids quantified as the fluorescence intensity of DCDFA (cytosol, n = 3 in technical duplicates, paired t-test) or Mitosox (mitochondrial, n = 3 in technical duplicates, paired t-test) normalized to haplogroup H. **f**) Cytosolic and Mitochondrial calcium levels of haplogroup H and J cybrids quantified as the fluorescence intensity of Fura Red (cytosol, n=7 in technical duplicates, paired t-test) or Rhod-2 (mitochondrial, n=5 in technical duplicates, paired t-test) normalized to haplogroup H.

a) KEGG Nicotinate and Nicotinamide Metabolism

gene	logFC	adj p-value
NADK	0.23	1.90E-04
NMNAT1	0.27	2.07E-03
NAMPT	0.09	2.19E-02
NT5M	0.24	4.59E-02
ENPP1	0.43	7.28E-02
NT5C	0.08	1.92E-01
NUDT12	0.05	5.56E-01
NMRK1	0.05	6.32E-01
NT5C3A	0.00	9.59E-01
NMNAT2	-0.02	8.94E-01
NADSYN 1	-0.10	9.11E-02
PNP	-0.09	3.15E-02
NNT	-0.32	3.36E-03
BST1	-0.79	5.24E-04
NNMT	-2.25	3.19E-05
NT5E	-0.49	7.00E-07
AOX1	-1.61	2.41E-08
NT5C2	-0.50	1.75E-09

d) KEGG One Carbon Pool by Folate

gene	logFC	adj p-value
TYMS	0.31	1.54E-03
MTHFS	0.20	3.73E-03
MTR	0.18	5.75E-04
MTHFD1	0.09	1.70E-01
MTHFD2L	0.02	8.63E-01
ATIC	-0.01	8.16E-01
SHMT1	-0.03	5.68E-01
MTFMT	-0.08	3.76E-01
MTHFR	-0.13	1.52E-01
ALDH1L1	-1.93	5.15E-02
GART	-0.06	1.72E-02
DHFR	-0.41	5.18E-03
MTHFD1L	-0.22	3.13E-05
CBS	-0.16	5.13E-05
SHMT2	-0.39	2.48E-06
MTHFD2	-0.47	1.21E-06

b)

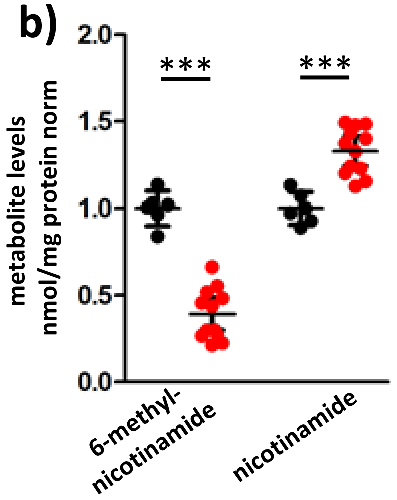

e) Targeted metabolomics 1C metabolism

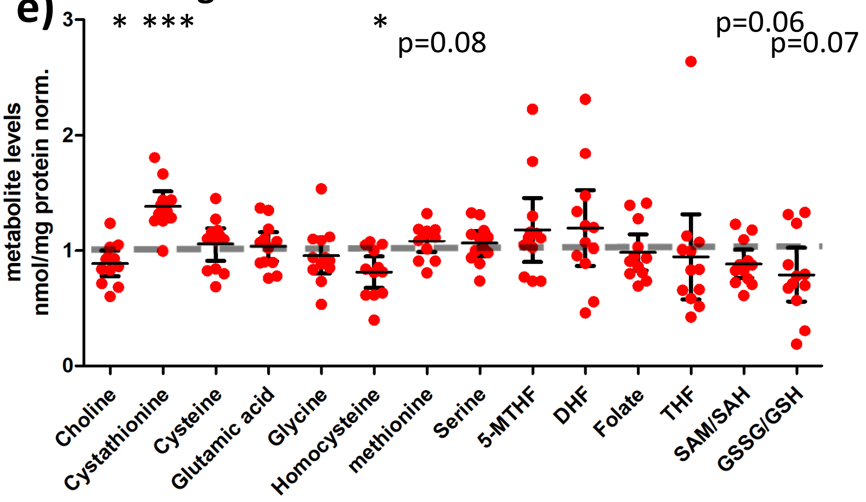

c) Targeted metabolomics organic acids

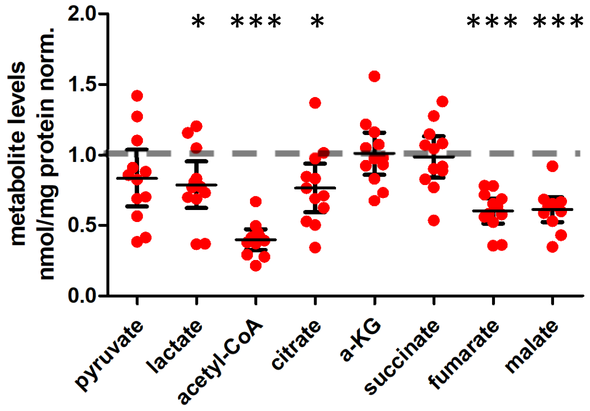

Fig.S2 *ND5* m.13708G>A-H7 mtDNA alters nicotinamide, TCA cycle, and one carbon metabolism

a) Differential gene expression between mutant and control cybrids for the KEGG Nicotinate and Nicotinamide Metabolism pathway. Log fold change (LogFC) is colored blue – white – red with blue indicating upregulation and red indicating downregulation of a gene in mutant cybrids compared to control. Adjusted (adj.) p-values are colored white – yellow with yellow indicating stronger significance.
b) Relative nucleotide levels in mutant cybrids normalized to control (n = 6/12 for control/mutant, unpaired t-test) detected in global metabolomics. **c)** Targeted metabolomics of TCA cycle intermediates in mutant cybrids normalized to control (n = 6 for control, n = 12 for mutant, One-sample t-test).
d) Differential gene expression between mutant and control cybrids for the KEGG One Carbon Pool by Folate pathway. Log fold change (LogFC) is colored blue – white – red with blue indicating upregulation and red indicating downregulation of a gene in mutant cybrids compared to control. Adjusted (adj.) p-values are colored white – yellow with yellow indicating stronger significance. **e)** Targeted metabolomics of one carbon metabolism in mutant cybrids normalized to respective control (n = 6 for control, n = 12 for mutant, One-sample t-test).

a) Upregulated modifications

modification	fold change	ratio	p-value
H3K4un	1.02	0.838	0.0003
H3.3K27unK36un	1.35	0.120	0.0006
H4K16ac	1.08	0.287	0.0058
H3K9me2	1.11	0.122	0.0083
H3K9unK14un	1.19	0.218	0.0087
H3K36me1	1.15	0.128	0.0177

b) Downregulated modifications

modification	fold change	ratio	p-value
H3K27me3	0.90	0.105	0.0003
H3K4me1	0.90	0.160	0.0003
H3K18acK23ac	0.78	0.041	0.0013
H3K18ac	0.80	0.043	0.0023
H3K9me3K14ac	0.91	0.016	0.0029
H3K4me3	0.83	0.001	0.0036
H3K4me2	0.84	0.001	0.0043
H3.3K27me3	0.70	0.201	0.0136
H3K9me1	0.84	0.321	0.0149
H3K9me1K14ac	0.89	0.141	0.0160
H3K27me3K36me1	0.84	0.012	0.0308
H4K20me1	0.76	0.332	0.0384

unmodified peptide

methylation

acetylation

methylation+acetylation

c) Single PTMs

modification	fold change	ratio	p-value
H4K16ac	1.07	0.444	0.0253
H3K9me2	1.07	0.161	0.0368
H3K36me1	1.05	0.261	0.1134
H4K20me2	1.23	0.383	0.1947
H4K20me3	1.35	0.005	0.1957
H3K9me3	1.04	0.083	0.2399
H3K9ac	1.47	0.004	0.3102
H3K4ac	2.05	0.000	0.5182
H3K27me1	1.01	0.107	0.8335
H3K23me1	0.99	0.001	0.9592
H3K27me2	0.99	0.419	0.8015
H3K18me1	0.93	0.001	0.7446
H4K12ac	0.98	0.185	0.7172
H3K36me3	0.94	0.057	0.6864
H4K5ac	0.96	0.067	0.6543
H3K36me2	0.87	0.027	0.5787
H3K23ac	0.96	0.339	0.4839
H4K8ac	0.96	0.111	0.4813
H3K14ac	0.96	0.271	0.1781
H3K27ac	0.84	0.003	0.1333
H4K20me1	0.76	0.332	0.0384
H3K9me1	0.86	0.462	0.0085
H3K4me2	0.84	0.001	0.0043
H3K4me3	0.83	0.001	0.0036
H3K18ac	0.79	0.084	0.0004
H3K4me1	0.90	0.160	0.0003
H3K27me3	0.90	0.117	0.0003

Fig.S3 *ND5* m.13708G>A-H7 mtDNA results in histone hypomethylation
a/b) Upregulated (a) and downregulated (b) post-translational modifications (PTMs) from histones H3 and H4 in mutant compared to control cybrids measured using mass spectrometry. The ratio indicates the fraction of the peptide with the respective modification, and the color-coding the type of modification present on the peptide, with blue being unmodified, red being methylation, yellow acetylation and orange methylation and acetylation. **c)** Single peptide modifications independent of the originating histone in mutant compared to control cybrids. Significant PTMs are colored according to their modification. Significance versus control was calculated for all PTMs using unpaired t-test (n=6).

a) Reactome Cholesterol Biosynthesis

gene	logFC	adj p-value
HSD17B7	0.63	6.91E-11
ACAT2	0.59	4.55E-10
DHCR7	0.64	8.25E-10
SQLE	0.48	2.16E-08
LSS	0.52	2.41E-08
NSDHL	0.40	2.57E-08
MSMO1	0.49	3.16E-08
FDFT1	0.56	4.39E-08
HMGCS1	0.72	8.91E-08
MVD	0.49	1.98E-06
MVK	0.48	2.81E-06
DHCR24	0.34	2.35E-05
CYP51A1	0.29	2.90E-04
FDPS	0.20	3.85E-04
TM7SF2	0.39	7.49E-03
SC5D	0.13	2.07E-02
HMGCR	0.27	2.69E-02
IDI1	0.06	1.61E-01
LBR	0.00	9.80E-01
IDI2		
PLPP6		
PMVK	-0.01	9.02E-01
EBP	-0.03	7.32E-01
GGPS1	-0.09	1.74E-01
ARV1	-0.15	1.20E-02

b) Reactome Ketone Body Metabolism

gene	logFC	adj p-value
AACS	0.35	1.27E-05
HMGCL	0.17	5.42E-03
BDH1	0.14	1.49E-02
HMGCLL1		
HMGCS2		
OXCT2		
ACAT1	-0.14	2.69E-03
BDH2	-0.31	1.69E-03
OXCT1	-0.26	7.61E-04
ACSS3	-0.91	1.35E-07

c)

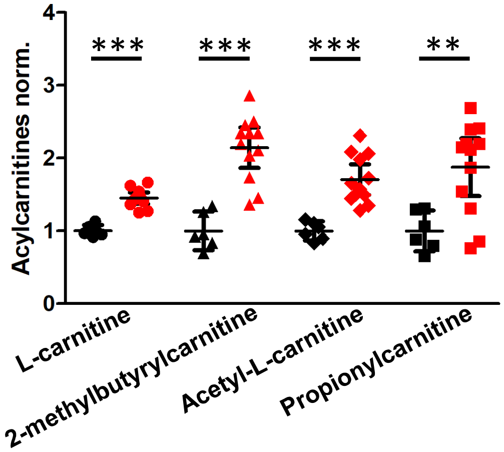

Fig.S4 *ND5* m.13708G>A-H7 mtDNA alters cholesterol, ketone and collagen metabolism
a/b) Differential gene expression between mutant and control cybrids for the Reactome cholesterol biosynthesis (a) and the Reactome Ketone Body Metabolism (b) pathway. Log fold change (LogFC) is colored blue – white – red with blue indicating upregulation and red indicating downregulation of a gene in mutant cybrids compared to control. Adjusted (adj.) p-values are colored white – yellow with yellow indicating stronger significance. **c)** Acylcarnitines detected in the global metabolomics screen in mutant cybrids normalized to control (n = 6 for control, n= 12 for mutant, unpaired t-test).

a) **Reactome Mitochondrial calcium ion transport**

gene	logFC	adj p-value
LETM1	0.20	8.90E-05
STOML2	0.19	4.06E-04
PMPCA	0.18	7.64E-03
PHB	0.15	2.23E-02
AFG3L2	0.12	2.62E-02
MICU2	0.07	7.27E-02
PARL	0.06	2.10E-01
PHB2	0.05	2.35E-01
PMPCB	0.04	3.63E-01
SLC8A3		
MAIP1		
MCUB		
MICU3	-0.10	1.85E-01
SLC8B1	-0.16	9.23E-02
AKAP1	-0.07	8.16E-02
SMDT1	-0.17	2.53E-02
SPG7	-0.28	6.48E-04
VDAC3	-0.55	6.22E-04
VDAC2	-0.28	1.58E-05
VDAC1	-0.41	1.53E-05
MCU	-0.35	5.44E-06
MICU1	-0.43	4.24E-07
YME1L1	-0.33	6.83E-09

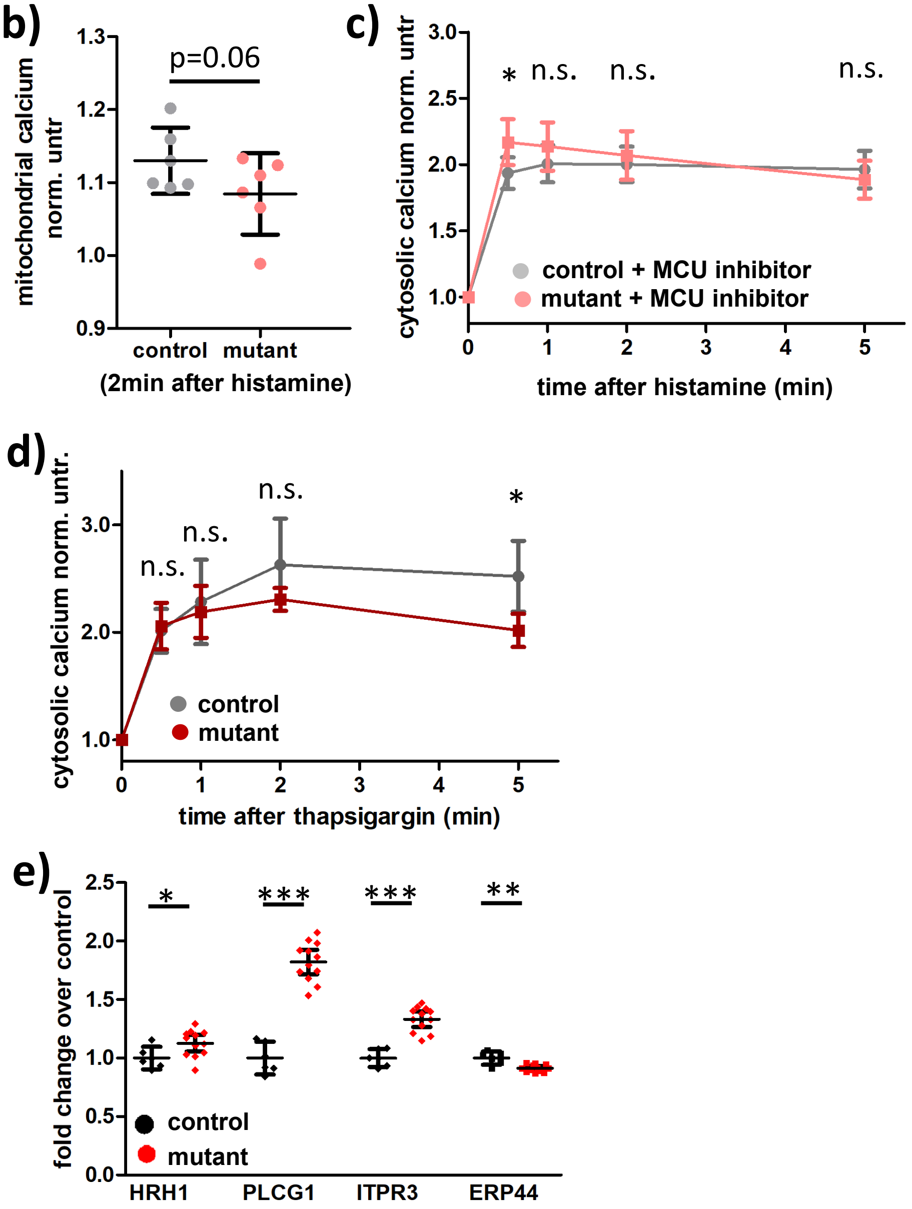

Fig.S5 *ND5* m.13708G>A-H7 mtDNA reduces mitochondrial calcium uptake and increases histamine sensitivity
a) Differential gene expression between mutant and control cybrids for the Reactome mitochondrial calcium ion transport pathway. Log fold change (LogFC) is colored blue – white – red with blue indicating upregulation and red indicating downregulation of a gene in mutant cybrids compared to control. Adjusted (adj.) p-values are colored white – yellow with yellow indicating stronger significance. **b)** Mitochondrial calcium levels in mutant and control cybrids 2 min after a 100 µM Histamine stimulus, normalized to calcium levels pre-histamine. Measured using Rhod-2 and flow cytometry (n = 6, each in technical duplicates, paired t-test). **c)** Cytosolic calcium levels in variant and control cybrids in response to a 100 µM histamine stimulus after pretreatment with MCU-inhibitor (10 µM KB-R7943), normalized to calcium levels pre-histamine (time point 0). Measured using Fura Red and confocal microscopy (n = 9, paired t-test). **d)** Cytosolic calcium levels in mutant and control cybrids in response to a 2 µM thapsigargin stimulus, normalized to calcium levels pre-thapsigargin (time point 0). Measured using Fura Red and confocal microscopy (n = 6, paired t-test). **e**) Differential gene expression between mutant and control cybrids for histamine 1 receptor (HRH1), phospholipase C (PLCG1) and IP3 receptor (ITPR3) and endoplasmatic reticulum protein 44 (ERP44), a negative regulator of IP3 receptor-mediated calcium release (n = 6 for control, n = 12 for mutant, unpaired t-test).

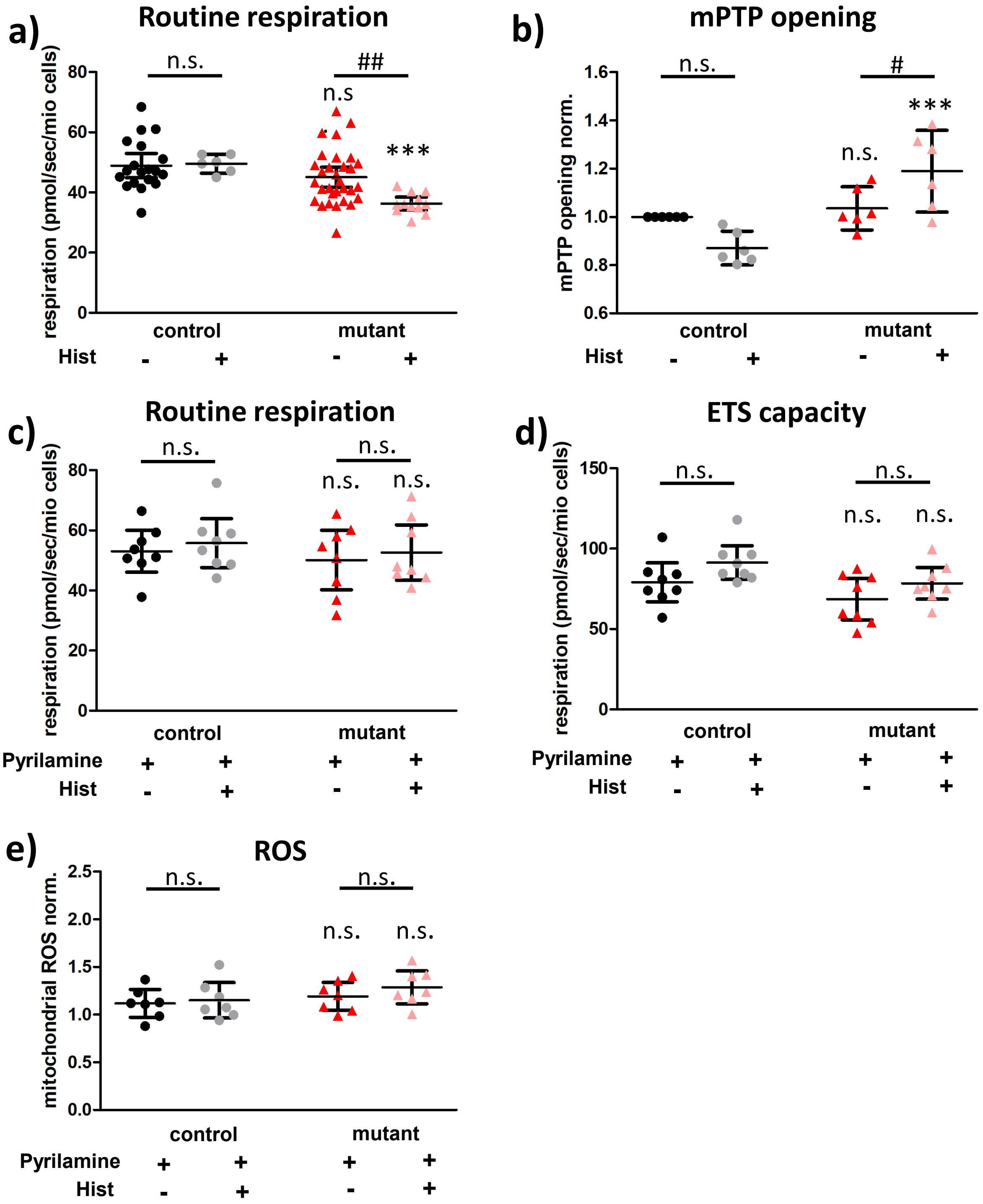

Fig.S6 Histamine toxicity in *ND5* m.13708G>A-H7 mtDNA cybrids is mediated via Histamine 1 receptor-mediated calcium release and mPTP opening

a) Routine respiration of intact control and mutant cybrids with and without pretreatment with 100 µM histamine for 24h measured using high-resolution respirometry (n = 19, 6, 31, 12 from left to right, each in technical duplicates, Kruskal Wallis test). **b)** mPTP opening in control and mutant cybrids with and without pretreatment with 100 µM histamine for 24h measured cobalt-calcein quenching in flow cytometry (n = 6, each in technical duplicates, One-Way ANOVA). **c/d)** Routine respiration (c) and electron transport system capacity (d) of intact pyrilamine-treated (100 µM) control and mutant cybrids with and without treatment with 100 µM histamine for 24 h measured using high-resolution respirometry (n=8, each in technical duplicates, Kruskal Wallis test). **e)** Mitochondrial ROS levels of pyrilamine-treated control and variant cybrids with and without treatment with 100 µM histamine for 24h quantified as the fluorescence intensity of Mitosox normalized to untreated control (n = 7, each in technical duplicates, One-way ANOVA). Significances between mutant and control are indicated by * and between treatment and control are indicated by #.

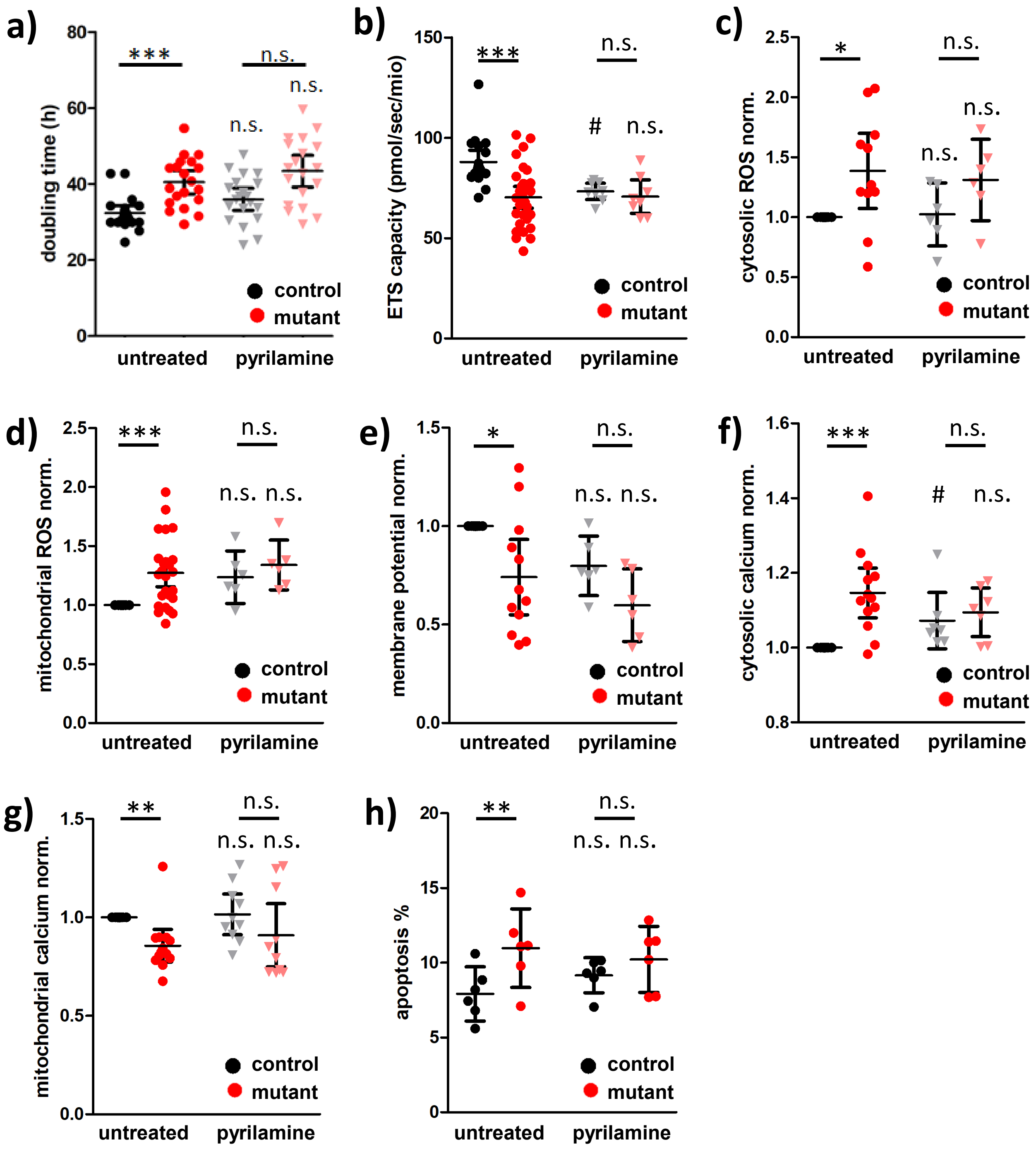

Fig.S7 Histamine 1 receptor inhibition reduced mitochondrial function but alleviates the negative effect of the *ND5* m.13708G>A-H7 mtDNA

Comparison of control and mutant cybrids untreated or cultured with 100 µM pyrilamine added to the medium. **a)** Doubling time (n=20, Kruskal Wallis test). **b)** Electron transport system capacity of intact cells (n = 19, 31, 8, 8 from left to right, each in technical duplicates, One-Way ANOVA). **c/d)** Cytosolic (c) and mitochondrial (d) ROS levels quantified as the fluorescence intensity of DCDFA (cytosol, n = 11, 11, 6, 6 from left to right, in technical duplicates, One-way ANOVA) or Mitosox (mitochondrial, n = 26, 26, 6, 6 from left to right, in technical duplicates, One-way ANOVA) normalized to untreated control. **e)** Mitochondrial membrane potential measured as the ratio of red to green fluorescence of JC-1 quantified by flow cytometry normalized to untreated control (n = 12, 12, 6, 6 from left to right, each in technical duplicates, One-way ANOVA). **f/g)** Cytosolic (f) and mitochondrial (g) calcium levels measured by flow cytometry using Fura Red (cytosol, n = 13, 13, 7, 7 from left to right, each in technical duplicates, Kruskal-Wallis test) or Rhod-2 (mitochondria, n = 13, 13, 10, 10, each in technical duplicates, Kruskal-Wallis test) normalized to untreated control. **h)** Apoptosis quantified as the fluorescence intensity of the cells after Annexin V staining using flow cytometry (n = 6, technical duplicates, Repeated measures ANOVA). Significances between mutant and control are indicated by * and between treatment and control are indicated by #.

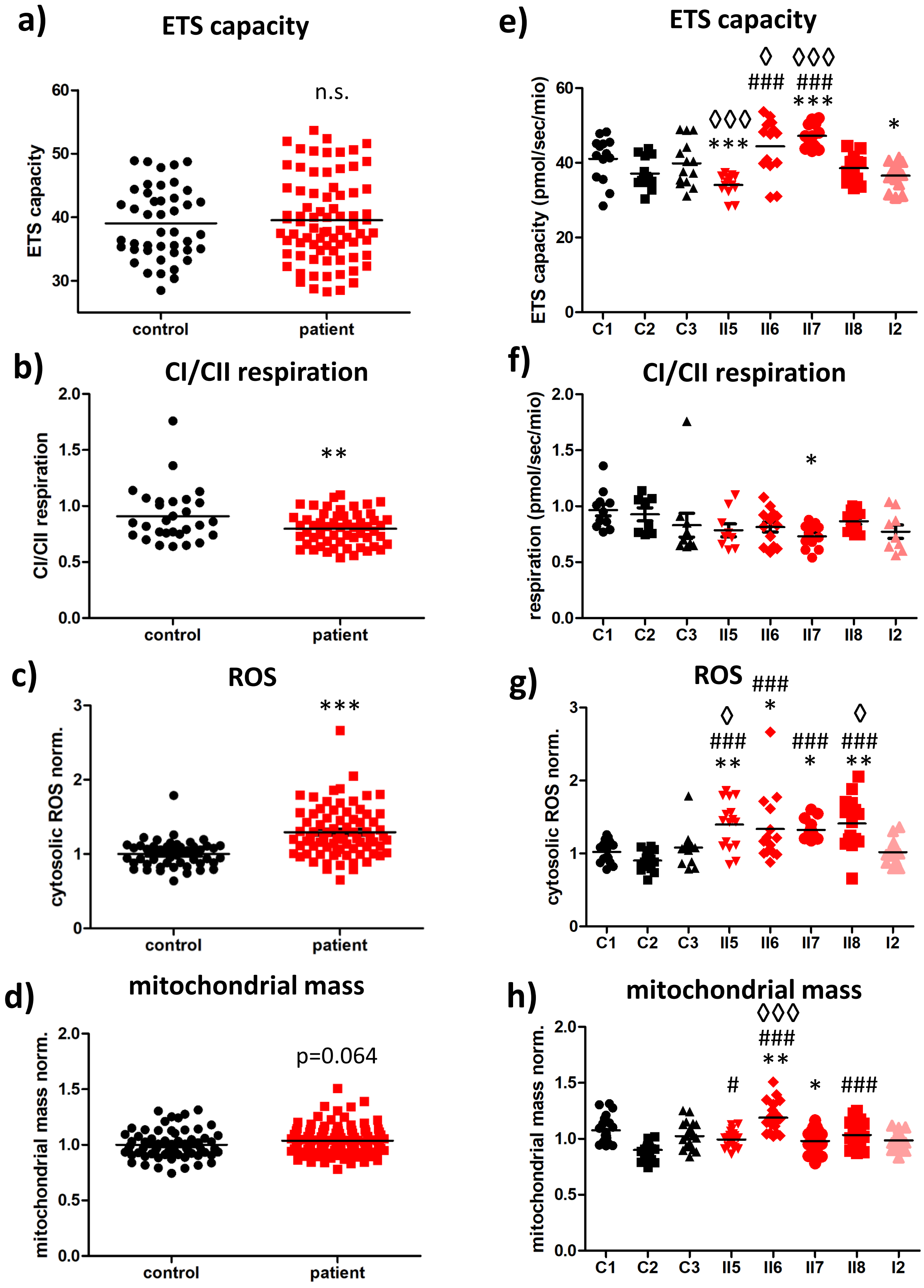

Fig.S8 Patient lymphoblasts show increased ROS levels and a switch from complex I to complex II respiration
Patient lymphoblastoid cells from the mother (I2) and 4 offspring (II5-II8) are compared to 3 haplogroup-matched controls. **a/e)** Electron transport system capacity in intact control and patient lymphoblastoid cells (n = 14). **b/f)** Complex I/complex II respiration in permeabilized lymphoblastoid cells (n= 11,8,10,13,13,9,12,9) **c/g)** Cytosolic ROS levels in control and patient lymphoblastoid cells quantified as the fluorescence intensity of DCDFA normalized to control (n = 16,16,15,15,14,16,15,16). **d/h)** Mitochondrial mass of control and patient lymphoblastoid cells quantified as fluorescence intensity of Mitotracker CMX ROS or Mitotracker Deep Red in flow cytometry normalized to control (n = 11). Significance for patient versus control (a-d) was calculated using unpaired t-test and significances for individual patients versus individual controls (e-h) were calculated using One-way ANOVA or Kruskal Wallis test. Significances relative to control are indicated by * for C1, # for C2, and ◊ for C3.

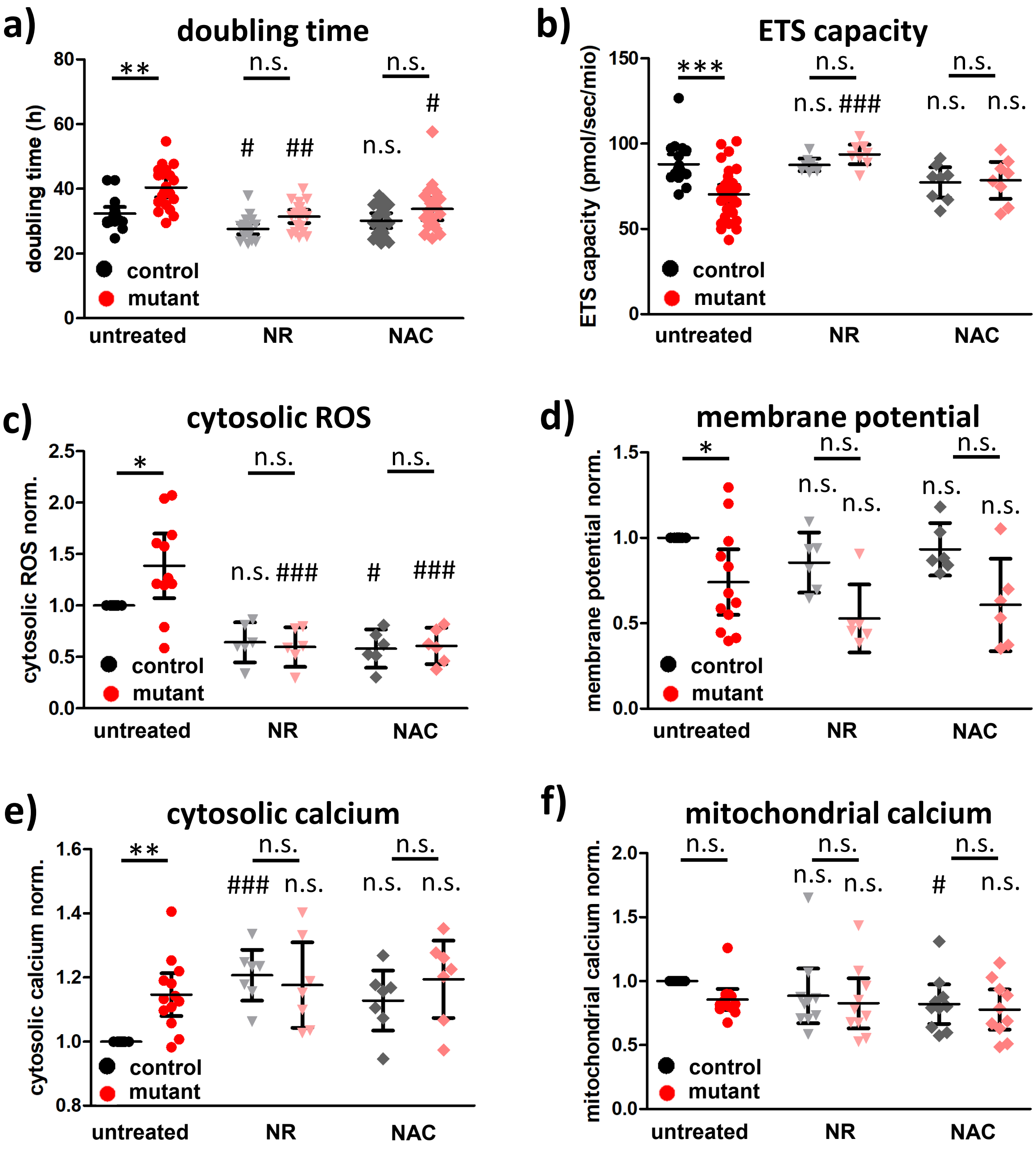

Fig.S9 NR and NAC can partially rescue the mitochondrial defect in the *ND5* m.13708G>A-H7 mtDNA cybrids

Comparison of control and mutant cybrids untreated or cultured with either 300 µM nicotinamide riboside or 1 mM N-acetylcysteine added to the medium. **a)** Doubling time (n = 20, Kruskal Wallis test). **b)** Electron transport system capacity of intact cells (n = 19, 31, 8, 8, 8, 8 from left to right, each in technical duplicates, One-Way ANOVA). **c)** Cytosolic ROS levels quantified as the fluorescence intensity of DCDFA (cytosol, n = 11, 11, 6, 6, 6, 6 from left to right, in technical duplicates, One-way ANOVA) normalized to untreated control. **d)** Mitochondrial membrane potential measured as the ratio of red to green fluorescence of JC-1 quantified by flow cytometry normalized to untreated control (n = 12, 12, 6, 6, 6, 6 from left to right, each in technical duplicates, One-way ANOVA). **e/f)** Cytosolic (e) and mitochondrial (f) calcium levels measured by flow cytometry using Fura Red (cytosol, n = 13, 13, 7, 7 from left to right, each in technical duplicates, Kruskal-Wallis test) or Rhod-2 (mitochondria, n = 13, 13, 10, 10, each in technical duplicates, Kruskal-Wallis test) normalized to untreated control. Significances between mutant and control are indicated by * and between treatment and control are indicated by #.

SI References

1. Ercan-Sencicek, A.G., et al., *L-histidine decarboxylase and Tourette's syndrome.* N Engl J Med, 2010. **362**(20): p. 1901-8.
